# Age- and sex-stratified prevalence of obstructive sleep apnea and stroke risk comorbidities: A large cross-sectional EHR study

**DOI:** 10.1371/journal.pone.0357824

**Published:** 2026-09-30

**Authors:** Rupesh Agrawal, Dursun Delen, Bruce Benjamin, Rani Misra

**Affiliations:** 1 Department of Health Informatics, College of Informatics, Northern Kentucky University, Highland Heights, Kentucky, United States of America; 2 Department of Management Science and Information Systems, Center for Health Systems Innovation, Spears School of Business, Oklahoma State University, Stillwater, Oklahoma, United States of America; 3 Department of Management Information Systems, Faculty of Business Administration, Haliç University, Istanbul, Türkiye; 4 Center for Health Sciences, Oklahoma State University, Tulsa, Oklahoma, United States of America; 5 Independent Researcher, San Antonio, Texas, United States of America; Karolinska Institutet, SWEDEN

## Abstract

**Objective:**

To characterize age- and sex-stratified prevalence patterns of obstructive sleep apnea and co-morbid conditions corresponding to ${\text{CHA}}_{2}{\text{DS}}_{2}-\text{VASc}$ components using a large, population-based electronic health record dataset.

**Study Design:**

The study cohort included 556,474 unique de-identified OSA patients (ICD-9 code 327.23), mined from HIPAA-compliant Cerner ${\text{HealthFacts}}^{®}$ database (1999–2016). The patient records were mined using Structured Query Language (SQL), stratified by age, gender, and stroke risk comorbidities per ${\text{CHA}}_{2}{\text{DS}}_{2}-\text{VASc}$, to conduct a population-based cross-sectional epidemiological study.

**Methods:**

Clinical and epidemiological research informatics methodologies are applied to characterize OSA prevalence patterns across age (5-year intervals), gender, and stroke risk comorbidities per ${\text{CHA}}_{2}{\text{DS}}_{2}-\text{VASc}$ criteria. Body-level organ system involvement was examined according to the Major Diagnostic Category (MDC) classification.

**Results:**

Age-stratified cross-sectional analyses showed that prevalence rates for OSA (m: 10.43%, f: 9.28%), hypertension (m: 7.37%, f: 7.11%), along with clinical encounters, peaked at age 55 for both genders, whereas the highest prevalence of cardiovascular comorbidities was observed in older age groups (ages 65–70). Hypertension was most prevalent (57.83%), followed by diabetes (34.7%), congestive heart failure (17.7%), atrial fibrillation (13.31%), vascular disease (12.3%), and prior stroke/TIA (4.78%).

MDC analysis revealed that prevalence is pronounced in ENT, cardiovascular, and musculoskeletal body systems, consistent with the pathophysiology of chronic intermittent hypoxia in OSA patients. These patterns reflect population-level age-specific prevalence differences rather than longitudinal disease progression.

**Conclusion:**

This large cross-sectional study (556,474 patients) provides detailed age- and sex-specific prevalence estimates of OSA-associated stroke-risk comorbidities. While causal or temporal inferences cannot be drawn, the findings highlight population-level prevalence patterns that may inform hypothesis-driven longitudinal studies and future evaluation of stroke-risk models.

## Introduction

Prevalence of obstructive sleep apnea (OSA) has been on the rise, and approximately 80% population is undiagnosed, and 1.36 billion adults globally suffer from OSA, where roughly 30 million adults are in the US [1]. Prevalence estimates are inconsistent across demographics (age, gender, race, and ethnicity) [2]. Additionally, patients with OSA exhibit a higher comorbidity burden with a sharp rise in OSA-related cardiovascular mortality rate [3,4]. However, traditional epidemiology and randomized controlled trials (RCTs) are scarce and lack statistical significance to establish strong guidelines for treatment. Clinical-decision gold standards, such as clinical trials, have proven time-consuming, expensive, and insufficient in terms of external validity, sample size, and overall significance [5,6]. Growing volumes of digital patient health information (PHI) and advances in AI-enabled scribe technologies can alleviate the clinical research burden and expedite RCTs [7,8].

Clinical Research Informatics (CRI) to patient health information (PHI) offers an alternative to overcoming the limitations of current gold standards. It produces scalable evidence where clinical trials are infeasible for a multitude of reasons, such as time, cost, validity, sample size, and significance [9]. Thus, EHRs should be considered complementary evidence to produce desired statistical support [10].

This study applies CRI through an evidence-based medicine (EBM) lens to address the following research question: “How is the Prevalence of OSA influenced by the patient’s age, gender, Stroke risk comorbidities, and major organ systems?” The study illustrates the utilization of non-clinical EHR data as a source of clinical information to uncover population-based patterns in age-trend prevalence estimates between the dependent variable (disease) and independent factors (comorbidity, age, and gender). Cardiovascular comorbidities (congestive heart failure, atrial fibrillation, and vascular disease) are analytically distinguished from metabolic risk factors (hypertension and diabetes). Although all are components of the ${\text{CHA}}_{2}{\text{DS}}_{2}-\text{VASc}$ framework, this separation allows clearer characterization of population-level prevalence patterns across related but clinically distinct disease categories.

## Materials and methods

### Digitization of healthcare and electronic health records

The American Recovery and Reinvestment Act (ARRA) of 2009 laid the foundation for the Health Information Technology for Economic and Clinical Health Act (HITECH) to organize patient health information to reduce disparities due to data fragmentation, biased clinical decision support, and the absence of EHR-based clinical interventions and epidemiological studies [11].

USFDA and PCORNET launched initiatives for “patient-centered and data-enabled clinical research to deliver results faster”. The goal is to support patients, caregivers, clinicians, and the broader healthcare community in a timely manner [12]. Government regulation and the resulting financial burden have motivated EHR adoption to achieve patient-centric clinical outcomes. Per data quality guidelines, “patient data should be correct and current.” [13]. The accumulation of petabytes of data has the potential to enable knowledge discovery through data mining and to digitize patients’ health [9]. EHR-driven clinical studies will be economical and timely than current studies, enabling clinical research to deliver results with greater significance [14].

### Evidence-based medicine (EBM)

The principle of evidence-based medicine (EBM) is the use of best practices for epidemiological evidence from observational studies (Cross-Sectional, Cohort, and Case-Control) and randomized controlled trials (RCTs). Evidence Level I is the highest level of evidence. Observational studies generate Level III and IV evidence. The validity, applicability, and level of evidence of clinical studies depend on the design, methodology, and cohort selection [15]. Gathering RCTs is an expensive, multi-year endeavor and risks losing financial support. Evidence has shown that well-designed observational studies can improve future RCTs [16,17]. Observational epidemiology studies based on EHRs have gained support as they estimate longitudinal prevalence and extract causality, a drawback of traditional cross-sectional studies due to the lack of data on disease progression. Thus, the application of CRI offers a paradigm shift by delivering cost-effective, time-efficient, and robust evidence [18].

### OSA, an epidemic

OSA is a chronic disease; its prevalence, comorbidities, and economic burden are increasing globally [19]. Over 30 million American adults suffer from OSA [18], and the severity increases the mortality [20]. Industrialization has disrupted sleep hygiene. Blue light suppresses melatonin, an essential hormone for sleep. Dietary habits have changed. Alcohol consumption decreases sleep quality, with an odds ratio (OR) of 3.37 [21]. The prevalence has spread beyond personal hygiene and habits. A motorist with Obstructive Sleep Apnea (OSA) increases the risk of road accidents by 2.5 times and costs U.S. taxpayers over $300 billion [22]. A report published in 2010 for Harvard Medical School titled “The Price of Fatigue: The Surprising Economic Costs of Unmanaged Sleep Apnea” stated that the economic cost of OSA is over $165 billion, and the financial burden is higher than asthma, heart failure, stroke, and hypertensive disease.

The established clinical guidelines and the understanding of the relationship between OSA and comorbidities are fragmented. Consequently, OSA is under-diagnosed and under-treated, with significant health and economic implications [23]. OSA is sleep-disordered breathing (SDB), characterized by repetitive episodes of a complete or partial upper airway collapse. The collapse of the airway leads to cessation of breathing or ineffective respiratory efforts, resulting in intermittent hypoxia (decreased oxygen in the body) and sleep disruption. Continuous positive airway pressure (CPAP) is considered the current gold standard for the treatment of OSA [24]. Low adherence to CPAP increases the OSA-multimorbidity and adds complexity to OSA care management.

### OSA and complexity

The International Classification of Sleep Disorders (ICSD-2) classifies 80 distinct sleep disorders. The heterogeneous phenotype and impact of OSA on multiple human organ systems (Fig 1) are further nuanced by complex relationships with demographic factors, such as age and gender [25], thus complicating the development of clinical guidelines for the diagnosis and treatment of OSA.

**Fig 1:**
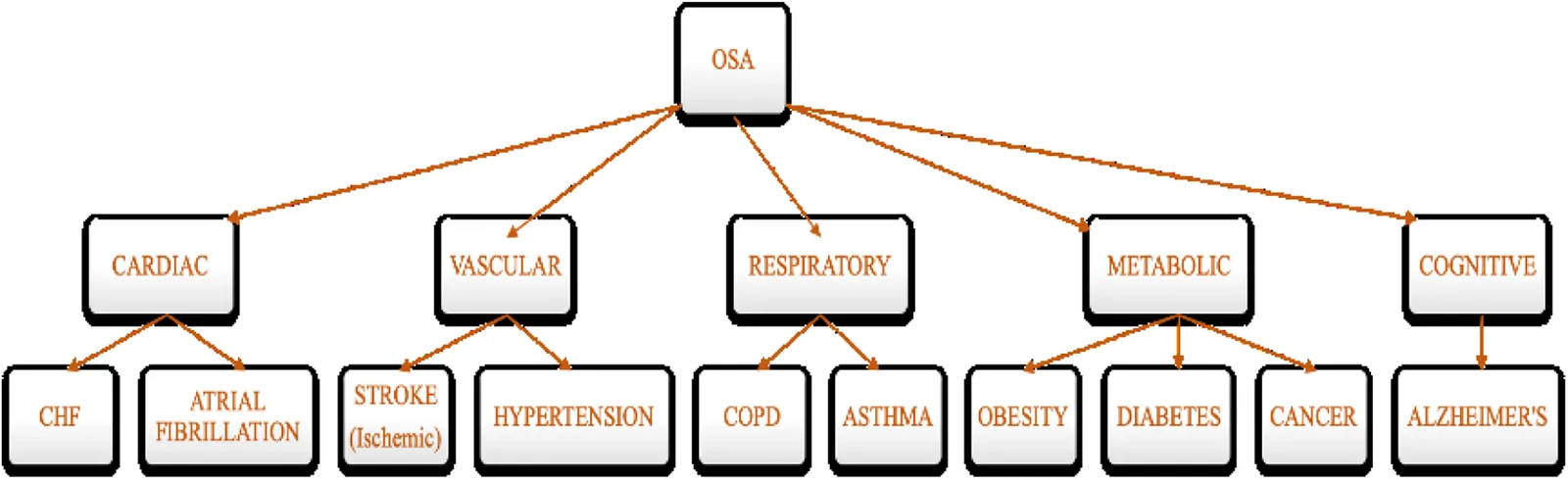
Obstructive sleep apnea and comorbidity.

### OSA and complex phenotype

The OSA phenotype is heterogeneous and affects vital organ systems (Refer to Fig 1) with a growing prevalence across age, gender, and geographic regions. OSA comorbidity increases mortality risk by as much as 140% [26]. The connection between OSA-AF is well established, and OSA-AF comorbidity condition is attributed to ischemic stroke, where stroke is the fourth leading cause of death with increasing mortality rate [27]. There is mounting evidence in support of the clinically relevant correlation between stroke, atrial fibrillation (AF), and obstructive sleep apnea (OSA) [27]. Researchers have endorsed including OSA as an independent factor in the stroke risk stratification scheme (RSS), i.e., ${\text{CHA}}_{2}{\text{DS}}_{2}-\text{VASc}$ [28]. However, OSA-Comorbidity complexity prevents establishing OSA as an independent causal factor of stroke risk, and OSA is not considered a risk factor in stroke risk stratification.

### Age and gender

Research data spanning multiple studies shows that up to 90% of men and 78% of women increase with age and peak among adults 55–59 years [29], and Men show a higher prevalence across all ages [30]. The presence of comorbidity further complicates the Prevalence and severity of OSA: Men with OSA are more susceptible to the presence of diabetes and heart disease, whereas women with OSA are more prone to hypertension [31].

### Epidemiology and limitations

Conventional OSA-epidemiology studies have had a narrow scope, qualitative symptom-based inclusion criteria such as snoring, gasping for air, and silent breathing pauses during sleep, and targeted patients with highly susceptible chronic comorbidities such as hypertension, rather than representative population-based sampling. Thus, traditional studies produced non-generalizable, varying, and inconsistent prevalence estimates [32]. Limitations due to measurement errors, limited inclusion/exclusion criteria, prone to selection bias, and site-specific variations in epidemiological studies produced limited evidence in support of prevalence, etiology, morbidity, and mortality. Thus, they are not able to postulate causal mechanisms of disease [32].

### Current gaps and future research

A large number of epidemiological studies have estimated the prevalence of OSA. However, the lack of comprehensiveness led to high variation and inconsistent conclusions [33]. Limited study design, lack of definition, inconsistent inclusion/exclusion criteria, selection bias, and reduced longitudinal subject participation resulted in unreliable validity and precision. A generalizable result is eclipsed by age, gender, and multi-morbidity [30]. Tables 1 and 2 show significant variation in the prevalence estimates across extant studies (% prevalence, age, gender, population, and diagnosed patient sample). It is safe to conclude that there is significant disagreement among studies regarding prevalence estimates of OSA and its comorbidities.

**Table 1:** Prevalence of stroke risk comorbidity.

Reference	Year	Comorbidity	Prevalence
Chen et al.	2025	Stroke	~62%
McKee et al.	2019	Stroke	30–90%
Huang et al.	2024	Hypertension	50–71%
Ahmad et al.	2001	Hypertension	30–83%
Li et al.	2024	Diabetes	50–75%
Jehan et al.	2018	Diabetes	18–87%
Tang et al.	2023	Congestive Heart Failure	34.80%
Khattak et al.	2018	Congestive Heart Failure	47–81%
Zhuang et al.	2025	Atrial Fibrillation	37%
Linz et al.	2018	Atrial Fibrillation	21–74%

**Table 2:** Prevalence of OSA, population, age, and gender.

Reference	Year	Population (Total)	Population (Male) (N, % Prevalence)	Population (Female) (N, % Prevalence)	Age	Country
Kline et al.	2025	5,320	2,718, 39%	2,602, 26%	≥18	USA
Wang et al.	2022	4,512	2,286, 34%	2,226, 21%	35–75	China
Senaratna et al.	2017	1,613	748, 23%	865, 17%	≥18	Australia
Heinzer et al.	2015	2,121	1,062, 50%	1,059, 23%	40–85	Switzerland
Franklin et al.	2013	10,000	N/A, N/A	400, 17%	20–70	Sweden
Appleton et al.	2013	220,000	64, 6%	400, 2%	≥18	Australia
Nakayama-Ashida et al.	2008	466	322, 18%	N/A, N/A	23–59	Japan
Huang et al.	2008	60,197	1,738, 3%	669, 1%	0–99	Australia
Sharma et al.	2006	2,400	88, 5%	63, 2%	30–60	India
Ip et al.	2004	3,074	153, 4%	106, 2%	30–60	Hong Kong
Udwadia et al.	2004	658	250, 8%	N/A, N/A	35–65	India
Kim et al.	2004	5,020	309, 5%	148, 3%	40–69	Korea
Durán et al.	2001	2,148	325, 3%	235, 3%	30–70	Spain
Bixler et al.	1998	4,364	741, 3%	N/A, N/A	20–100	USA
Young et al.	1993	3,513	352, 4%	250, 2%	30–60	USA

### Disease classification

In 1990, the ICSD developed codes for diagnostic and epidemiological purposes for sleep disorders, such as OSA [34]. Consequently, epidemiological studies lacked agreement on prevalence estimates (Table 2, Fig 2) due to differences in criteria for OSA, including snoring, EDS, fatigue, choking, and the oxygen de-saturation index (ODI). The current study addresses the gap by using diagnostic codes (ICD-9: 327.23; ICD-10: G47.33) to ensure accurate patient identification of OSA patients per the ICSD classification (Tables 3-6).

**Table 3:** Disease classification – MDC, DRG, ICD.

Major Diagnostic Code (MDC)	Disease Related Group (DRG)	International Classification of Diseases (ICD)
Single Organ System Groups	Products (Appendectomy)	Diseases and Procedures
1–23 (Principal Organ)	467 / 897 Groups	1,400 / 69,000 Codes
24 (Multiple Trauma)		
25 (HIV)		
26 (Transplants)		
27 (Ungroupable)		
Motivation: Cost calculations	Cost-based reimbursement	Generic disease classification
	“Grouper” program based on ICD diagnoses, procedures, age, sex, discharge status, and complications/comorbidities	Classifying diseases, nuanced classifications of signs, symptoms, abnormal findings, complaints, social circumstances, external causes
	Assumption: Patients are clinically similar	Morbidity/mortality statistics, reimbursement systems, automated decision support
Top Level Group	Subgroup of MDC	Subgroup of MDC and DRG

**Table 4:** MDC and ICD mapping for OSA (ICD-9 & ICD-10).

ICD-9: Diseases of the Nervous System (320–389)	ICD-10: Diseases of the Nervous System (G00–G99)
327 Organic disorders of initiating and maintaining sleep	G40–G47 Episodic and paroxysmal disorders
327.2 Organic sleep apnea, unspecified	G47 Sleep disorders
327.2 Organic sleep apnea NOS	G47.3 Sleep apnea
327.21 Primary central sleep apnea	G47.30 Sleep apnea, unspecified
327.22 High altitude periodic breathing	G47.31 Primary central sleep apnea
**327.23 Obstructive sleep apnea**	**G47.33 Obstructive sleep apnea (adult/pediatric)**
327.24 Idiopathic sleep-related non-obstructive alveolar hypoventilation	G47.34 Idiopathic sleep-related non-obstructive alveolar hypoventilation
327.25 Congenital central alveolar hypoventilation syndrome	G47.35 Congenital central alveolar hypoventilation syndrome
327.26 Sleep-related hypoventilation/hypoxemia	G47.36 Sleep-related hypoventilation in conditions classified elsewhere
327.27 Central sleep apnea with another medical condition	G47.37 Central sleep apnea in conditions classified elsewhere
327.29 Other organic sleep apnea	G47.39 Other sleep apnea

**Table 5:** DRG–ICD mapping: Obstructive sleep apnea (G47.33, 327.23).

DRG Code	Description
011	Tracheostomy for face, mouth & neck diagnoses or laryngectomy with MCC
012	Tracheostomy for face, mouth & neck diagnoses or laryngectomy with CC
013	Tracheostomy for face, mouth & neck diagnoses or laryngectomy without CC/MCC
154	Other ear, nose, mouth & throat diagnoses with MCC
155	Other ear, nose, mouth & throat diagnoses with CC
156	Other ear, nose, mouth & throat diagnoses without CC/MCC

Obstructive Sleep Apnea is grouped within MS-DRG v37.0.

**Table 6:** MDC to DRG mapping.

MDC	Description	DRG Range
0	Pre-MDC	001–017
1	Diseases and Disorders of the Nervous System	020–103
2	Diseases and Disorders of the Eye	113–125
3	Diseases and Disorders of the Ear, Nose, Mouth, Throat	129–159
4	Diseases and Disorders of the Respiratory System	163–208
5	Diseases and Disorders of the Circulatory System	215–316
6	Diseases and Disorders of the Digestive System	326–395
7	Diseases and Disorders of the Hepatobiliary System, Pancreas	405–446
8	Diseases and Disorders of the Musculoskeletal System and Connective Tissue	453–566
9	Diseases and Disorders of the Skin, Subcutaneous Tissue, Breast	573–607
10	Diseases and Disorders of the Endocrine, Nutritional, Metabolic System	614–645
11	Diseases and Disorders of the Kidney, Urinary Tract	652–700
12	Diseases and Disorders of the Male Reproductive System	707–730
13	Diseases and Disorders of the Female Reproductive System	734–761
14	Pregnancy, Childbirth, Puerperium	765–782
15	Newborn and Other Neonates (Perinatal Period)	789–795
16	Diseases and Disorders of the Blood and Blood Forming Organs and Immunological Disorders	799–816
17	Myeloproliferative DDs (Poorly Differentiated Neoplasms)	820–849
18	Infectious and Parasitic DDs (Systemic or unspecified sites)	853–872
19	Mental Diseases and Disorders	876–887
20	Alcohol/Drug Use or Induced Mental Disorders	894–897
21	Injuries, Poison, Toxic Effects of Drugs	901–923
22	Burns	927–935
23	Factors Influencing Health Status and Other Contacts with Health Services	939–951
24	Multiple Significant Trauma	955–965
25	Human Immunodeficiency Virus Infection	969–977
–	MDC Category Missing	981–989; 998, 999

**Fig 2:**
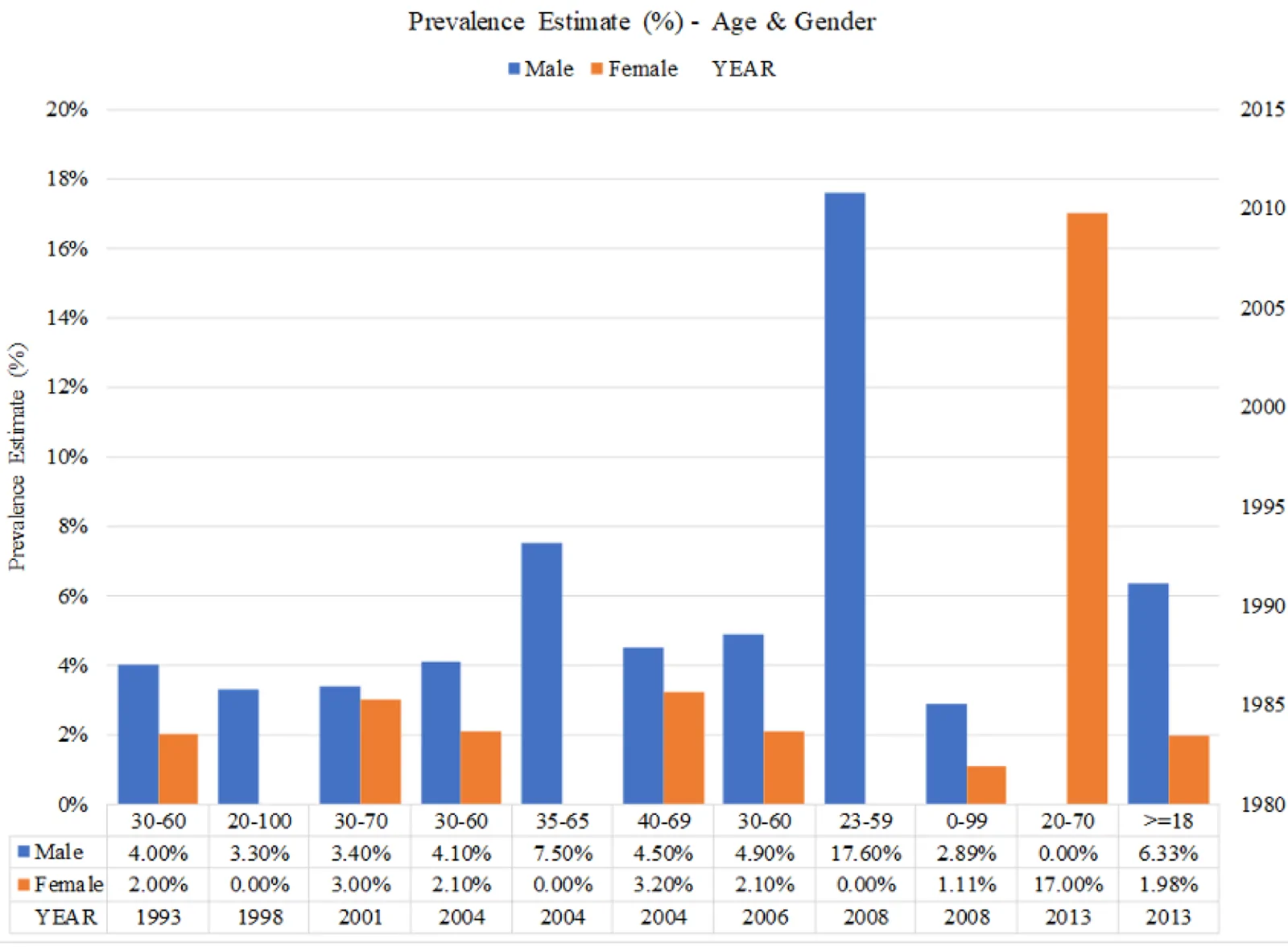
Prevalence estimate (%) – age vs gender.

### Major diagnostic code and drug-related group

Beyond ICD classification, Major Diagnostic Categories (MDC) and Diagnosis-Related Groups (DRG) organize ICD codes by organ system and related diseases. DRG classifies patients who are clinically similar in terms of diagnosis, treatment, length of hospital stays, and resource utilization for mapping prospective hospital payments. OSA is grouped within MDC 01 “Diseases and Disorders of the Nervous System/Sense Organs” (Table 6), enabling organ system-based epidemiological analysis.

### Clinical informatics with EHR for epidemiology

Clinical research informatics (CRI) harnesses big EHR data to extract epidemiological intelligence. CRI provides an accurate phenotype for complex diseases such as OSA by enabling precise clinical guidelines and overcoming challenges in RCTs.

This work represents the first in a series of two studies, with increasing levels of evidence, based on ICSD criteria on 60 million U.S. population-representative patient records to simulate comprehensive epidemiological evidence. Grounded in EBM principles, the current work establishes a foundation for examining the role of Obstructive Sleep Apnea (OSA) in stroke risk stratification.

### Study design & data source

The current study has two objectives: first, to characterize OSA prevalence patterns and compare these findings with the existing epidemiological literature. Second, to estimate OSA prevalence across stroke-risk comorbidity stratified by age and gender for refinement of the ${\text{CHA}}_{2}{\text{DS}}_{2}-\text{VASc}$ stroke-risk stratification scheme in patients with atrial fibrillation (AF). Differences observed across age groups represent population-level prevalence distributions and do not reflect longitudinal trajectories or temporal ordering of disease onset in individual patients. Fig 3 illustrates the study’s methodology.

**Fig 3:**
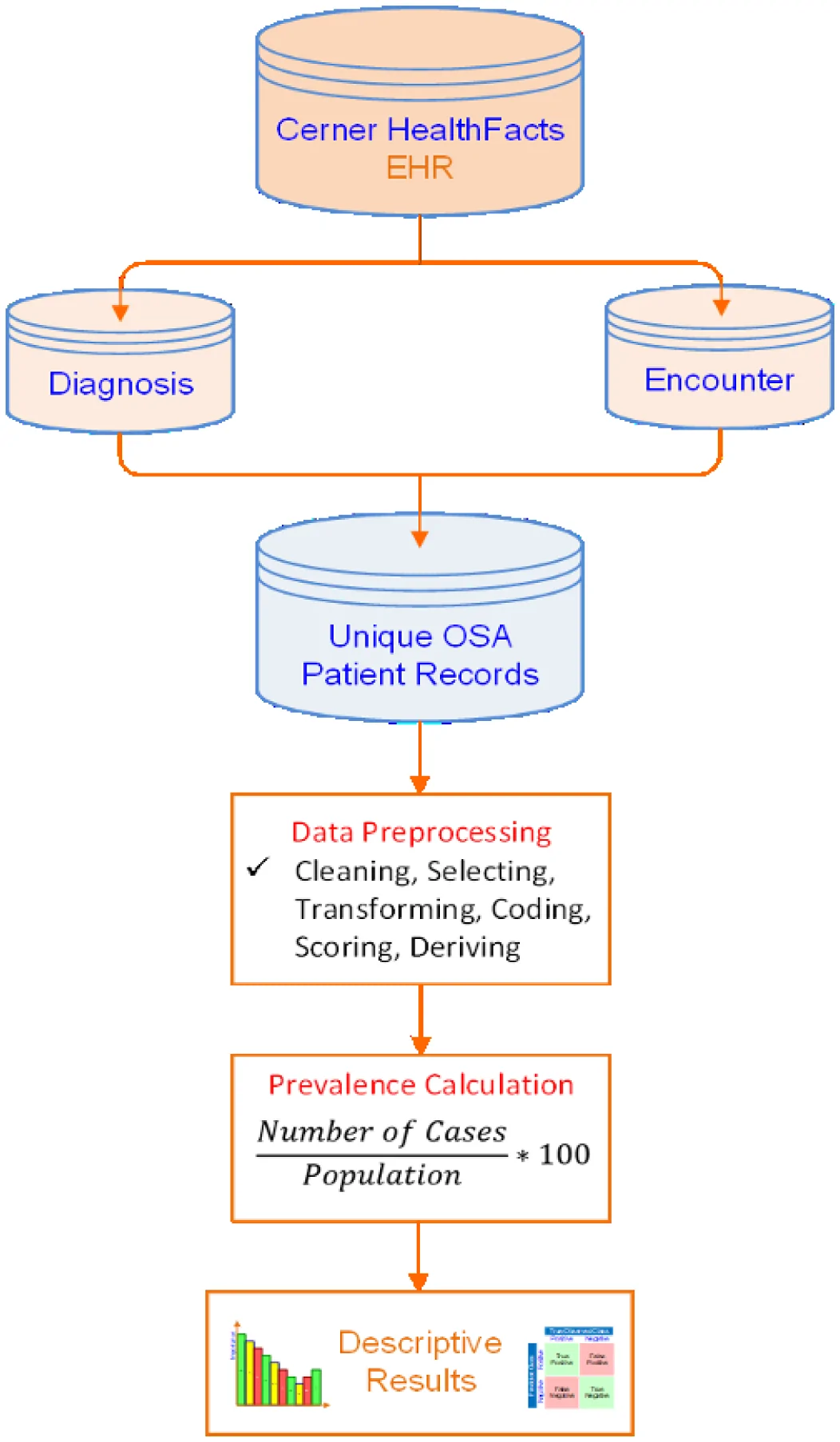
Research methodology.

The prevalence estimates were calculated as the “proportion of patients with OSA relative to the total number of patients in each stratification group.” The formula is written as follows:


$$\begin{array}{c} \text{Prevalence}=\frac{\text{Number of Patients in Sample with Characteristics}}{\text{Total Number of Patients in Sample}} \end{array}$$ (1)

The 2019 AHA/ACC/HRS guideline recommends using the ${\text{CHA}}_{2}{\text{DS}}_{2}-\text{VASc}$ risk stratification scheme (RSS) in assessing risk in patients with AF [35]. The risk of stroke in atrial fibrillation (AF) patients is 5–6 times [36]. The correlation between stroke, AF, and OSA is clinically significant [37], and research has endorsed OSA as a factor of [28] stroke risk. However, OSA-comorbidity complexity has prevented inclusion in the ${\text{CHA}}_{2}{\text{DS}}_{2}-\text{VASc}$ stratification scheme (RSS) [28]. The ${\text{CHA}}_{2}{\text{DS}}_{2}-\text{VASc}$ [28] is an acronym: congestive heart failure (CHF), Hypertension, age (≥75), Diabetes, history of stroke/transient ischemic attack/thromboembolic event, Vascular disease, Age 65–74 years, and sex category (female).

### Patient selection, data extraction, and classification

De-identified, HIPAA-compliant electronic health records (EHRs) from the Cerner ${\text{HealthFacts}}^{®}$ database (1999−2016) were mined using Structured Query Language (SQL) to identify unique OSA patients (ICD-9 code 327.23). The data for the research was accessed between 03/04/2018 and 30/07/2020. Fig 4 illustrates the breakdown of unique OSA patients (556,474) identified from 63 million patient records representing over 400 clinical encounters or visits. Extracted variables are $Patient_{Sk}$ (Unique Patient ID), *Diagnosis Type* (ICD-9), *Diagnosis Description*, *MDC Description*, and patient demographic variables (patient geographical location, Race, Age, Gender, and Marital Status). ${\text{CHA}}_{2}{\text{DS}}_{2}-\text{VASc}$ risk factors and comorbidities were identified using ICD-9 codes (Table 7). Data quality precautions were implemented prior to use for final analysis to address accuracy, redundancy, and missing values. Exclusion criteria included: removal of inconsistent coding (records marked both male and female), missing critical variables, duplicate encounters, and implausible values (e.g., age > 130 years).

**Table 7:** CHA_2_DS_2_-VASc risk factor & comorbidities.

Diagnosis	ICD-9	ICD-10
**Atrial Fibrillation**		
Atrial fibrillation and flutter	427.3	I48
Paroxysmal atrial fibrillation	427.3	I48.0
Persistent atrial fibrillation	–	I48.1
Chronic atrial fibrillation	–	I48.2
Unspecified atrial fibrillation and atrial flutter	–	I48.9
Unspecified atrial fibrillation	–	I48.91
**CHA**_**2**_ **DS**_**2**_ **-VASc Scheme**		
Hypertension	401.x, 402.x, 403.x, 404.x, 405.x	I10, I11, I12, I13, I15
Age ≥ 75 years	–	–
Diabetes mellitus	250.x	E10, E11, E12, E13, E14
Prior stroke or TIA or thromboembolism	434.x, 435.x	G45, I63
Vascular disease	440.2x, 410.x, 411.x, 412	I70.0, I70.2, I70.3, I70.4, I70.5
**Obstructive Sleep Apnea**		
Obstructive sleep apnea (adult/pediatrics)	327.23	G47.3x

**Fig 4:**
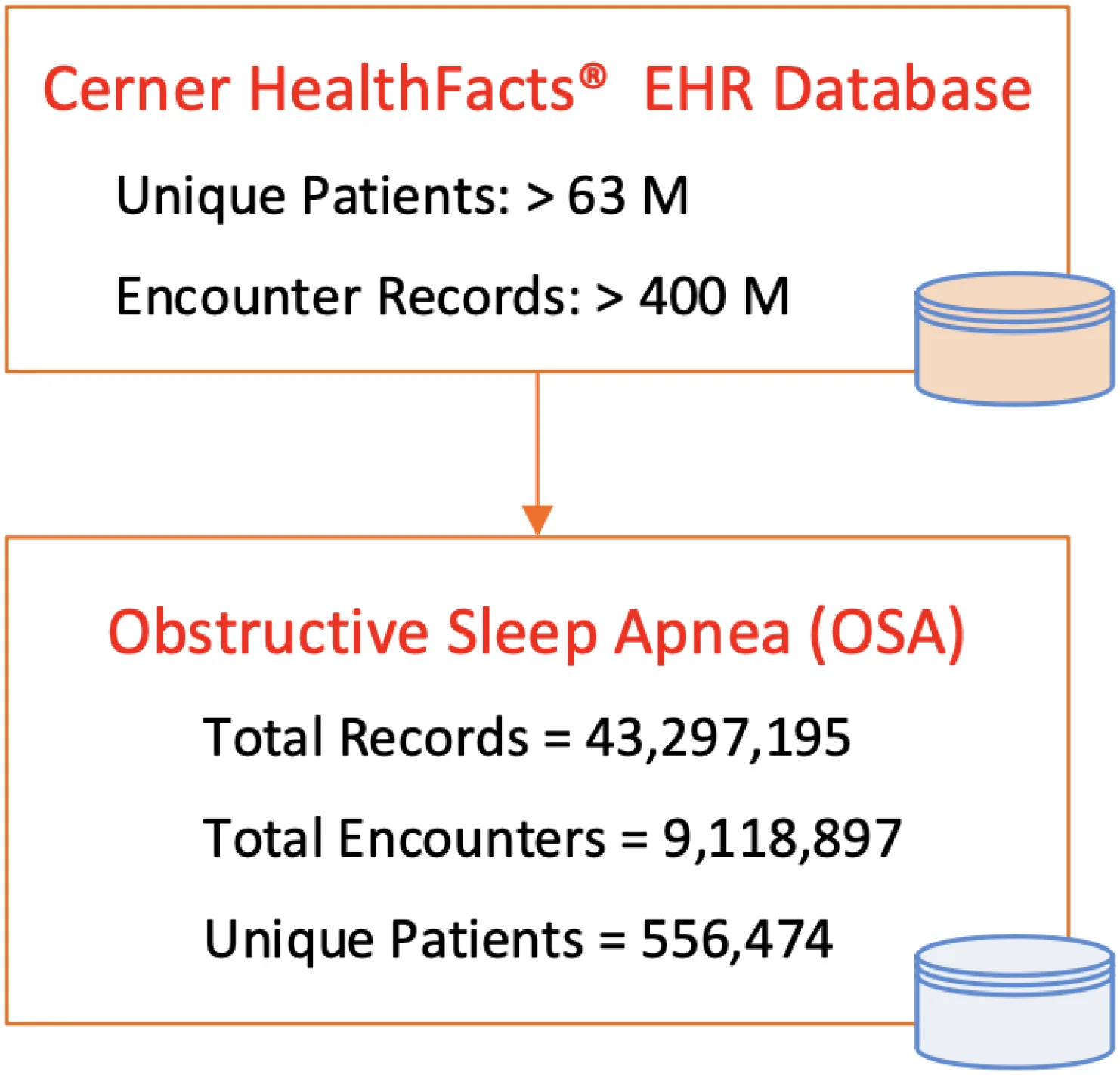
Patient population OSA and comorbidity.

### Ethics statement

This study utilized de-identified electronic health record data from the Cerner ${\text{HealthFacts}}^{®}$ database. All patient data were de-identified per HIPAA Safe Harbor standards prior to researchers’ access. No informed consent was required as subjects could not be identified or re-identified. Data were obtained under a data use agreement between Oklahoma State University and Cerner Corporation.

## Results

### OSA patient sample characteristics

The final cohort comprised 556,474 unique patients diagnosed with OSA (ICD-9: 327.23), including 312,450 males (56.1%) and 244,024 females (43.9%), with ages ranging from 18 to 95 years (mean age: 54.3 ± 14.2 years). Fig 4 summarizes the patient selection and cohort construction. Figs 5–7 describe the demographic distribution of the study cohort. The cohort was primarily Caucasian (67.71%), Urban (74.83%), and Married (44.95%).

**Fig 5:**
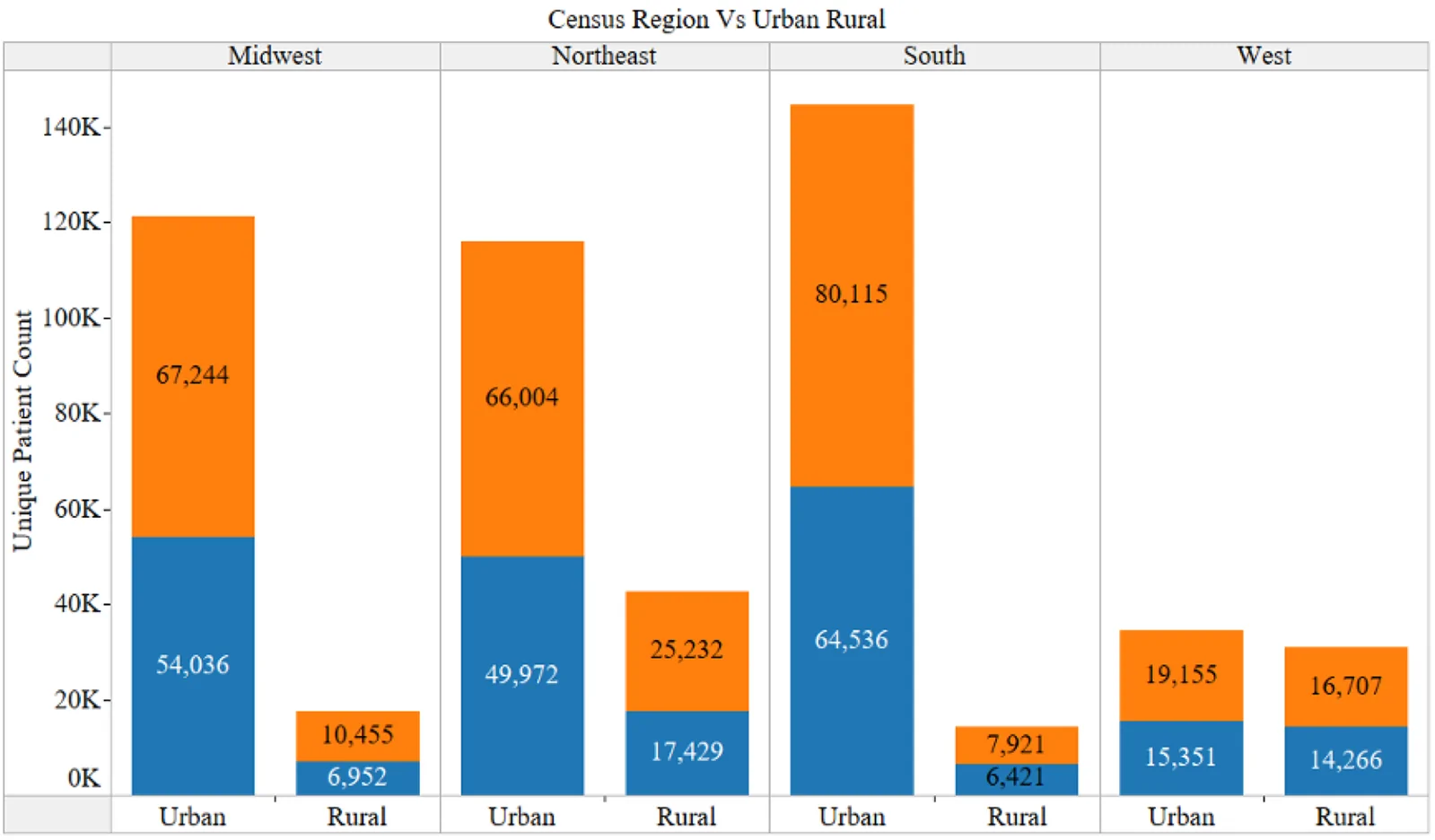
Population distribution – USA.

**Fig 6:**
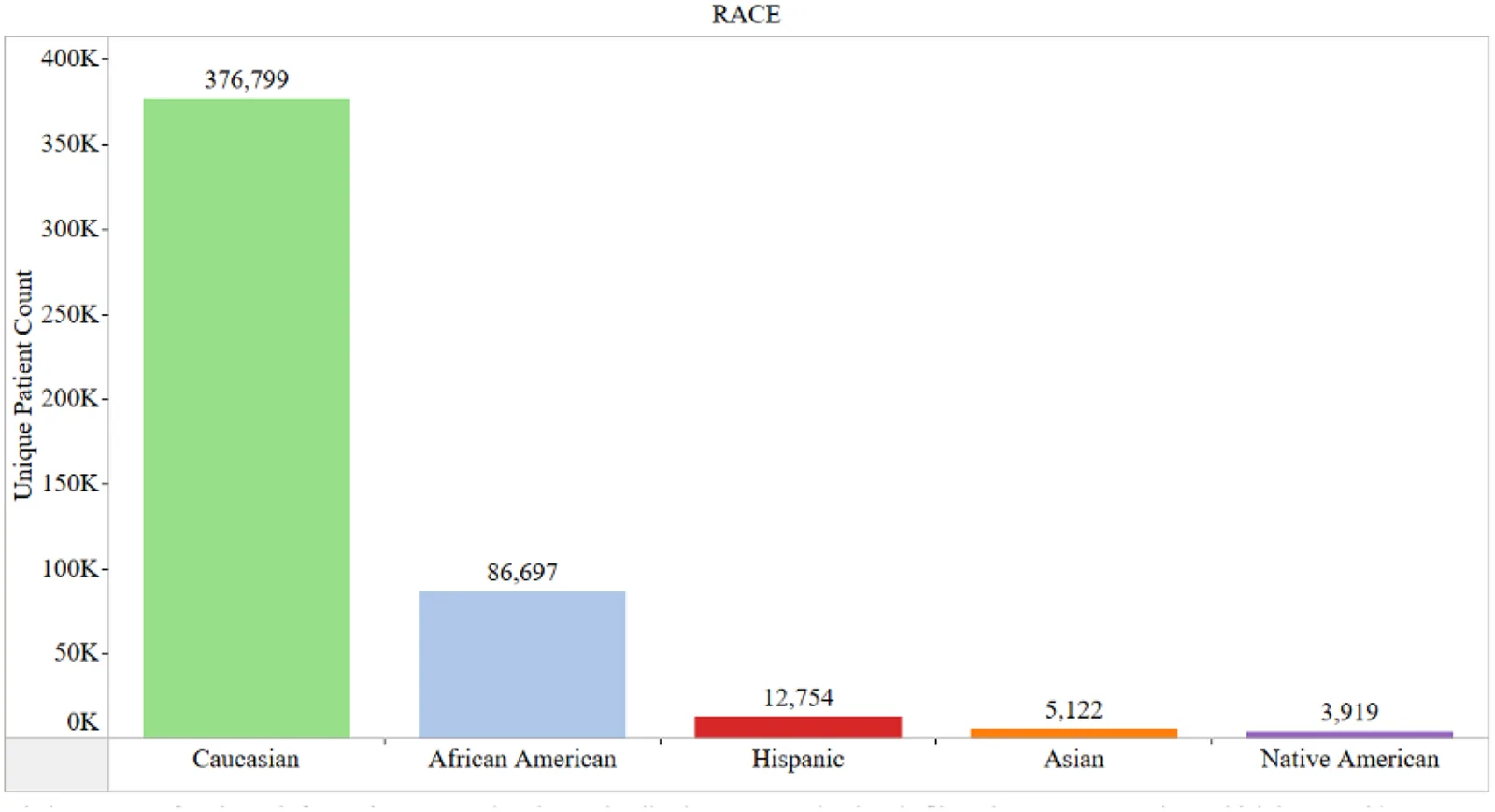
Population distribution – Race.

**Fig 7:**
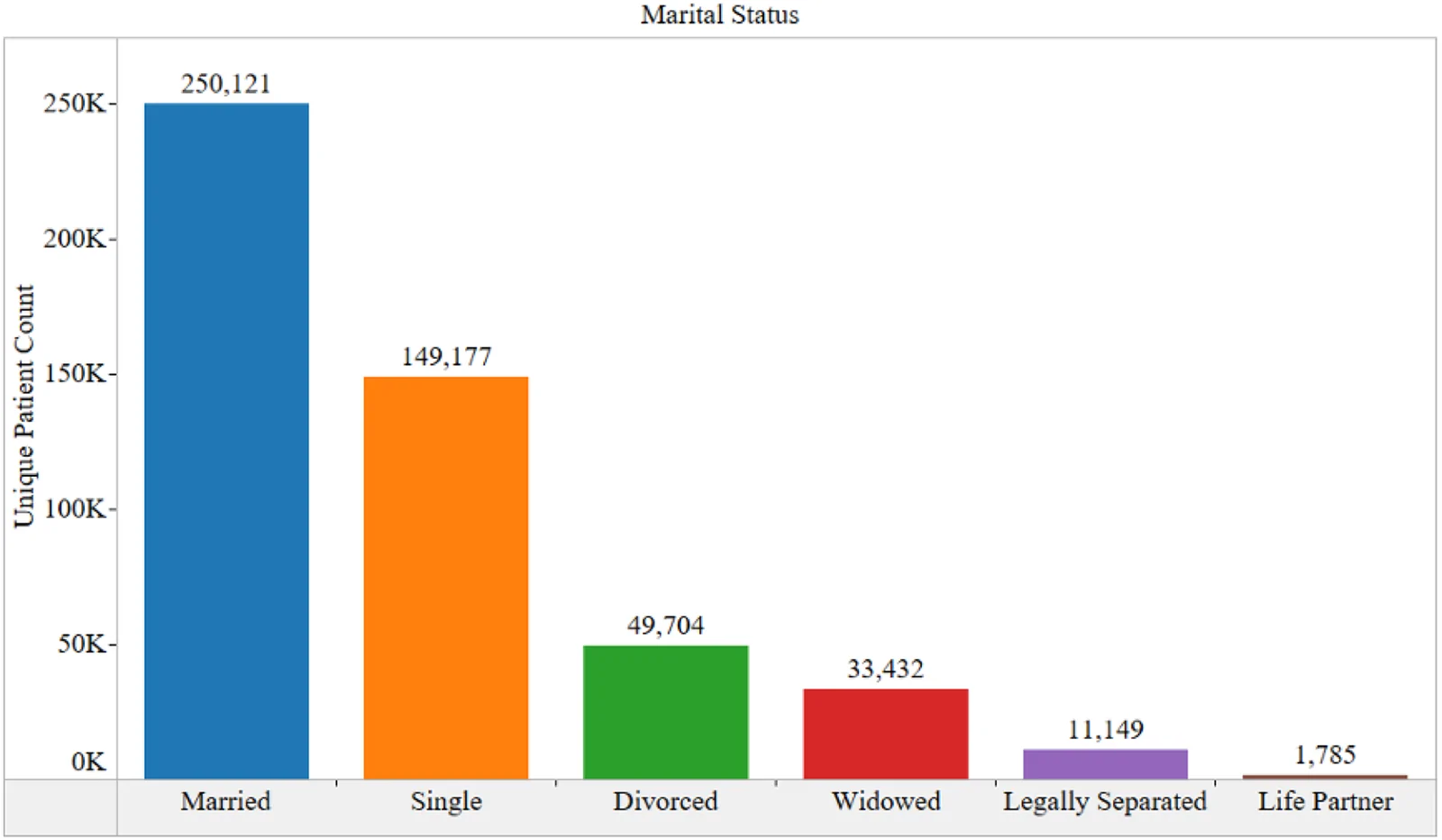
Population distribution – Marital status.

### OSA comorbidity prevalence

Refer to Fig 8 for a visual representation of the overall prevalence estimate for both genders stratified for stroke-risk comorbidities.

**Fig 8:**
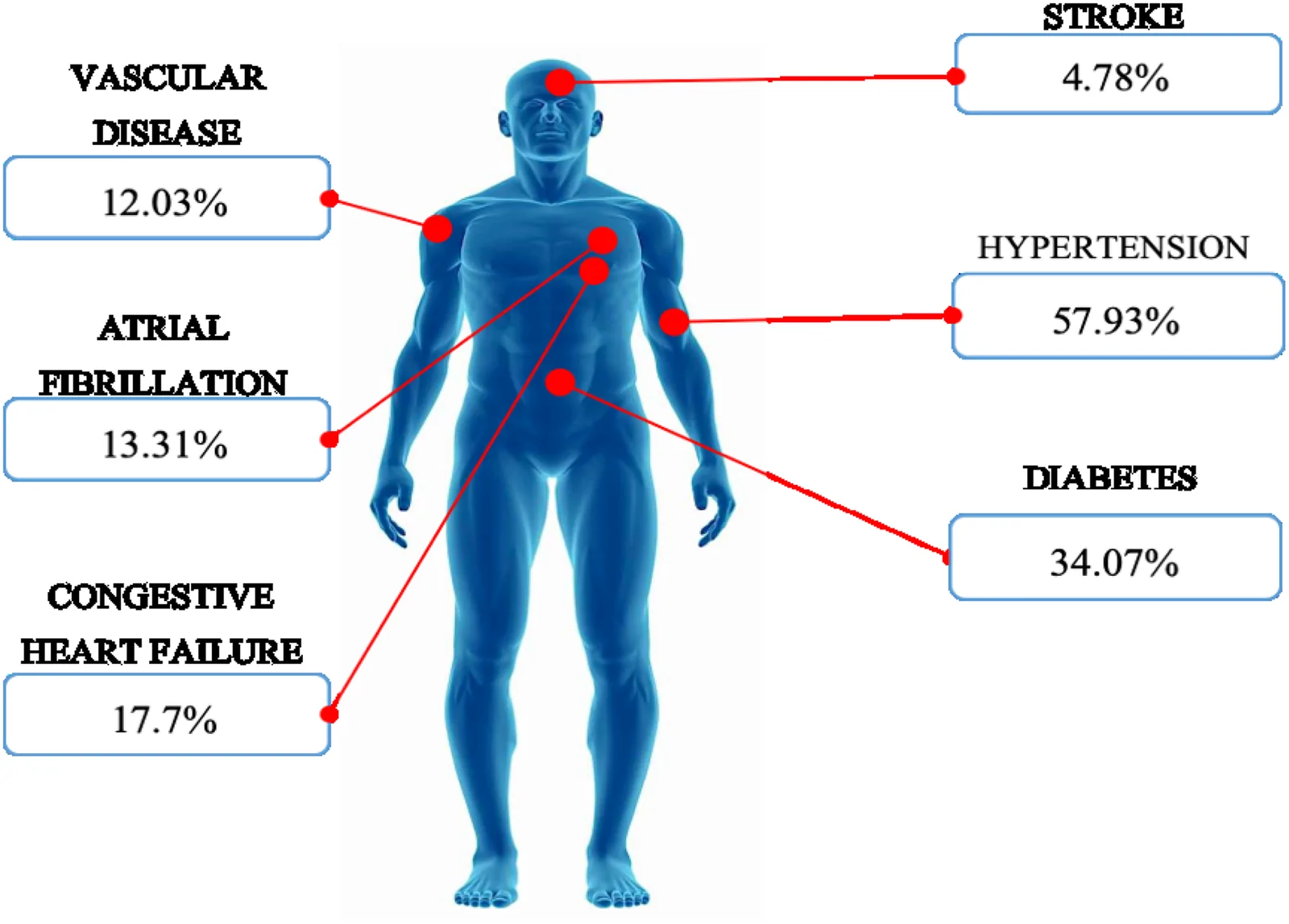
Comorbidity prevalence estimates (%) – OSA patients.

Among the OSA cohort, hypertension is the most prevalent comorbidity (57.83%, n = 321,811) with the second highest prevalence for diabetes (34.7%, n = 193,096), followed by congestive heart failure (17.7%, n = 98,496), atrial fibrillation (13.31%, n = 74, 067), vascular disease (12.3%, n = 68,446), and prior stroke/TIA (4.78%, = 26,599). Prevalence estimates for diabetes and hypertension are within the ranges reported in epidemiological studies; however, those for stroke, CHF, and AF are significantly lower than previously reported in the literature.

### OSA prevalence by age and gender

Healthcare utilization reached its peak at age 55, with females having higher utilization compared to males. OSA prevalence also peaked at age 55 for both sexes. For the female cohort (n = 244,024), peak prevalence was 9.28% (n = 22,645). Whereas for the male cohort (n = 312,450), peak prevalence was 10.43% (n = 32,589). Despite greater healthcare utilization among females, age-specific prevalence patterns were slightly higher among males than females (Fig 9).

**Fig 9:**
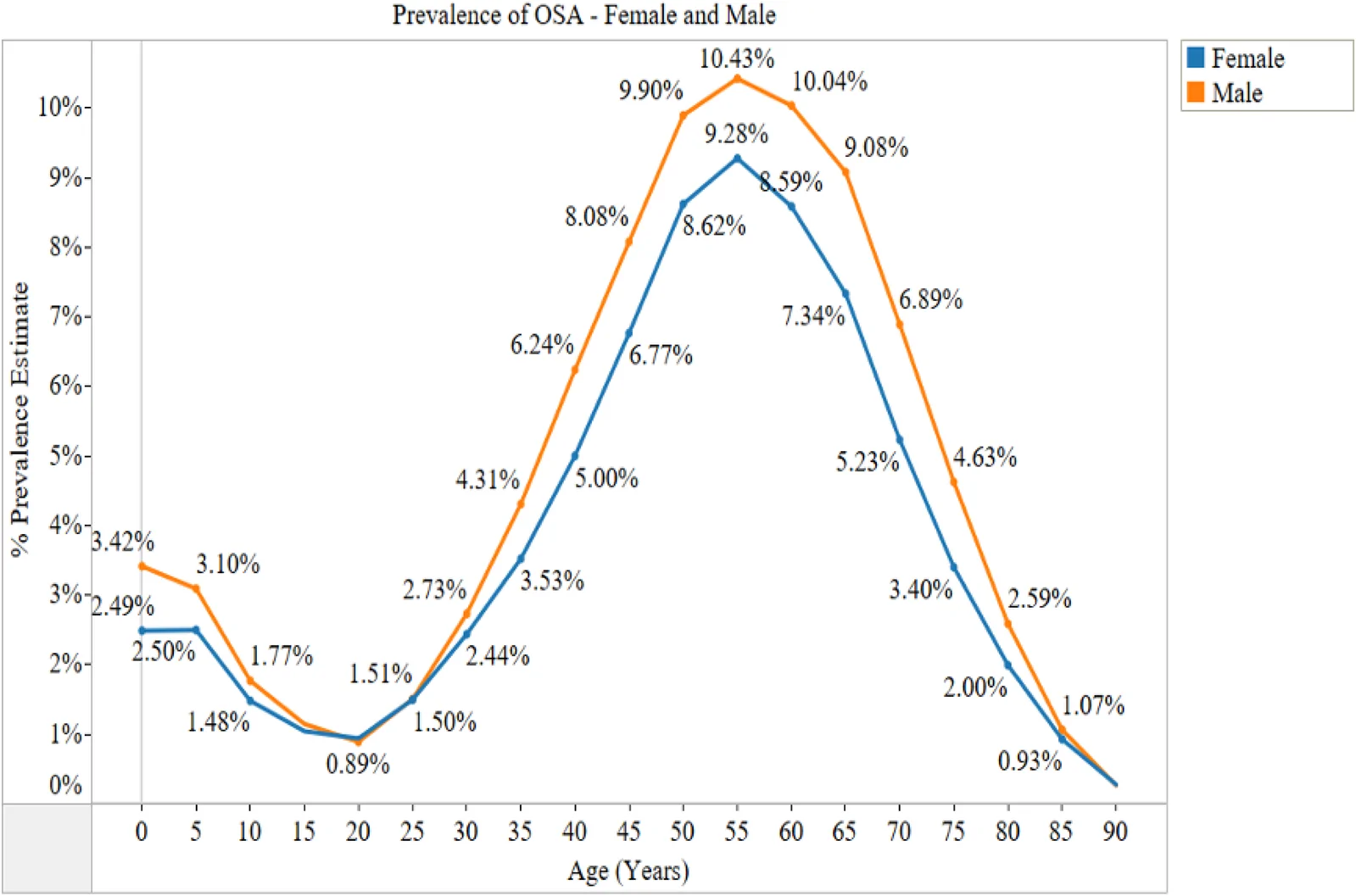
Prevalence estimate (%) – OSA.

### OSA comorbidity prevalence by age and gender

The current section summarizes the comorbidity prevalence by age and gender. The prevalence estimates for each comorbidity were calculated from OSA cohort (males: n = 312,450; females: n = 244,024).

Hypertension and diabetes showed similar prevalence rates between genders but peaked at different ages. Hypertension prevalence among OSA patients peaked at 55 years for both genders (Fig 10), with rates of 7.37% (n = 23,016) for men and 7.11% (n = 17,340) for women. Diabetes prevalence peaked at 60 years with peak prevalence rates for men 7.91% (n = 24,715), and for women 7.58% (n = 18,495) (Fig 11).

**Fig 10:**
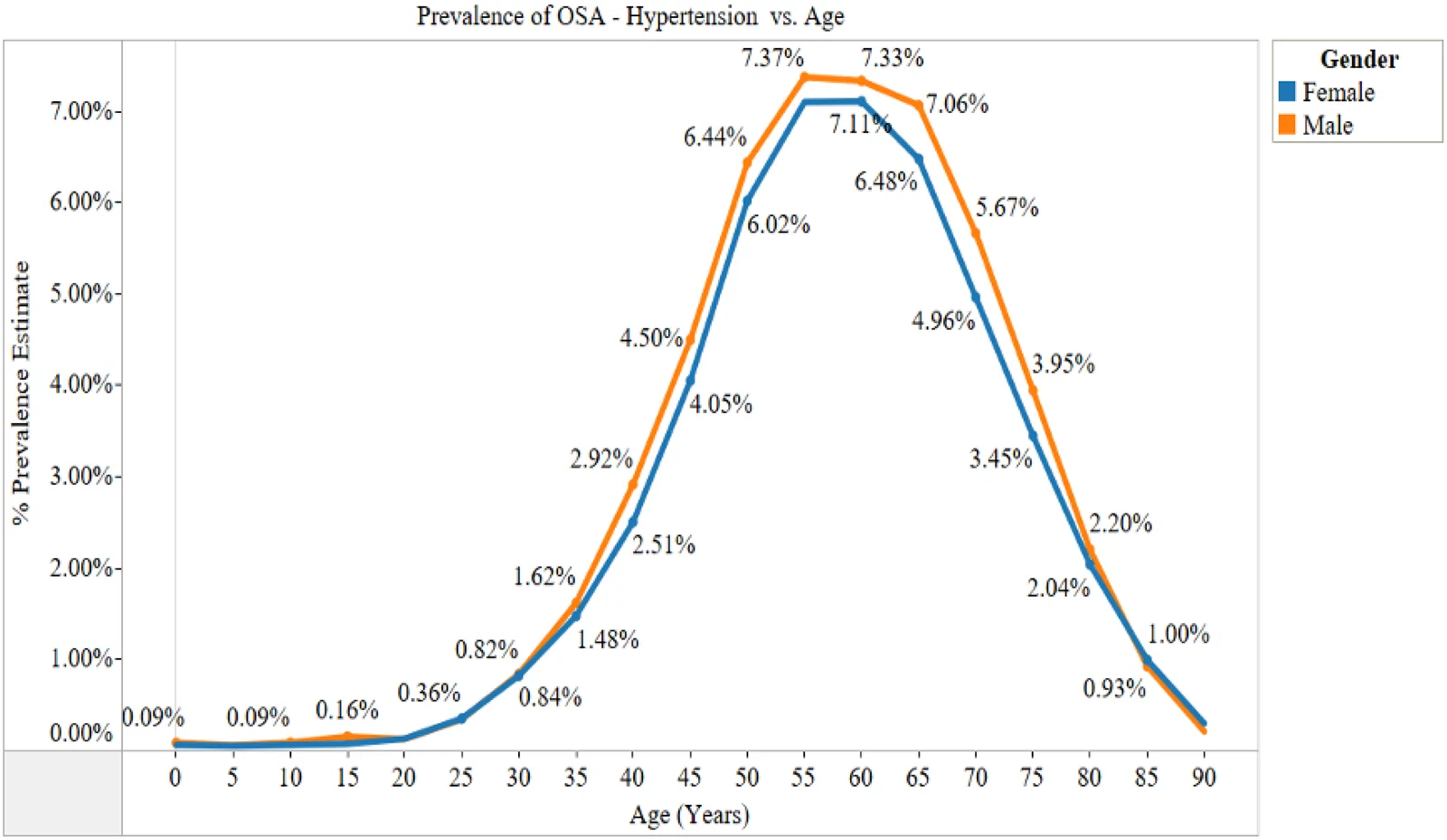
Prevalence estimate (%) – Hypertension.

**Fig 11:**
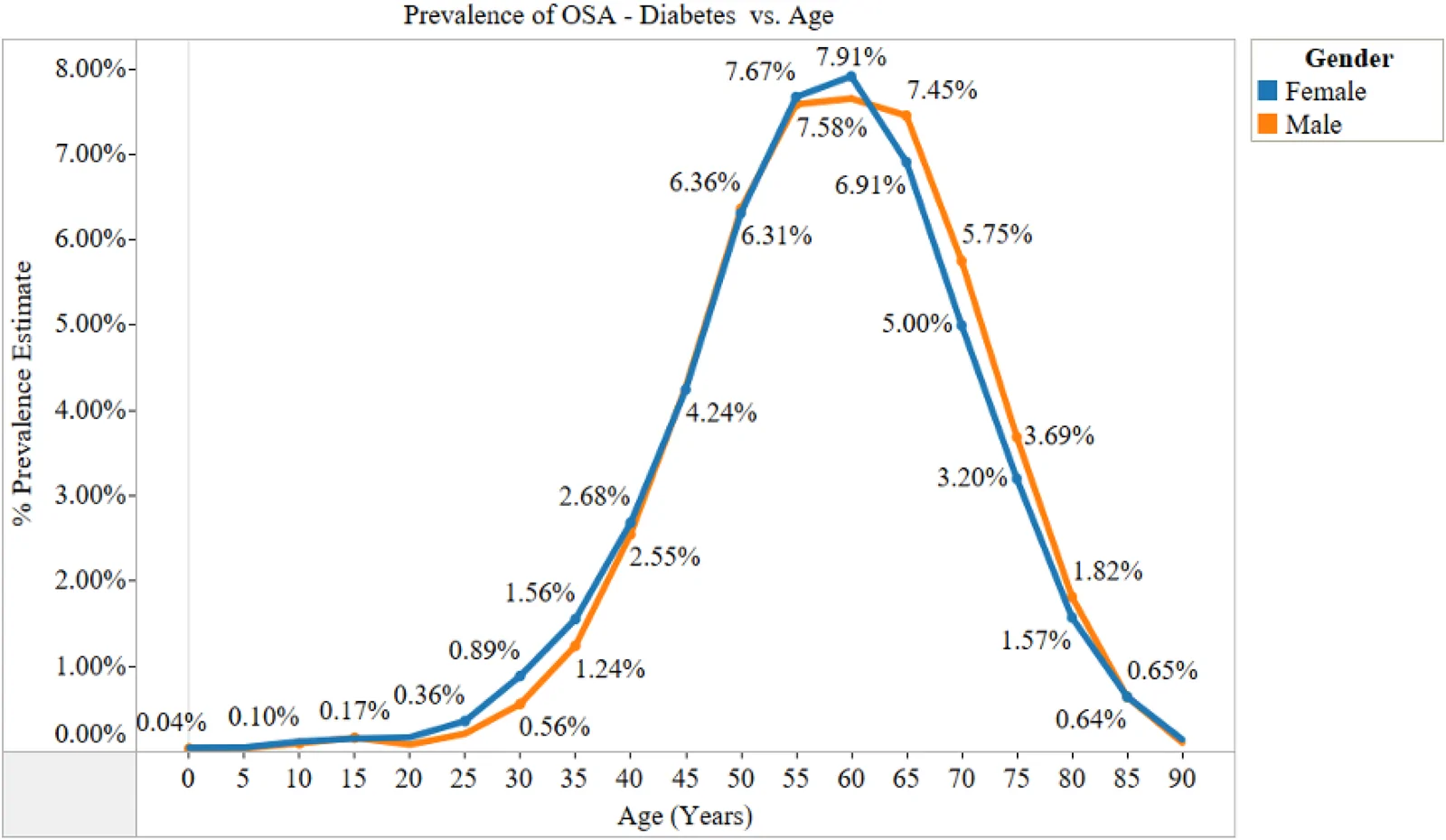
Prevalence estimate (%) – Diabetes.

Cardiovascular health or comorbidities demonstrated higher prevalence rates in males and peaked at older age (65–70 years) compared to hypertension (55 years). CHF prevalence among OSA patients peaked at 65 years for both genders (Fig 12), at 9.3% (n = 29,048) in men and 6.97% (n = 16,998) in women. AF prevalence peaked later at ages 70, with peak rates of 10.53% (n = 32,901) in men and 6.66% (n = 16,229) in women (Fig 13).

**Fig 12:**
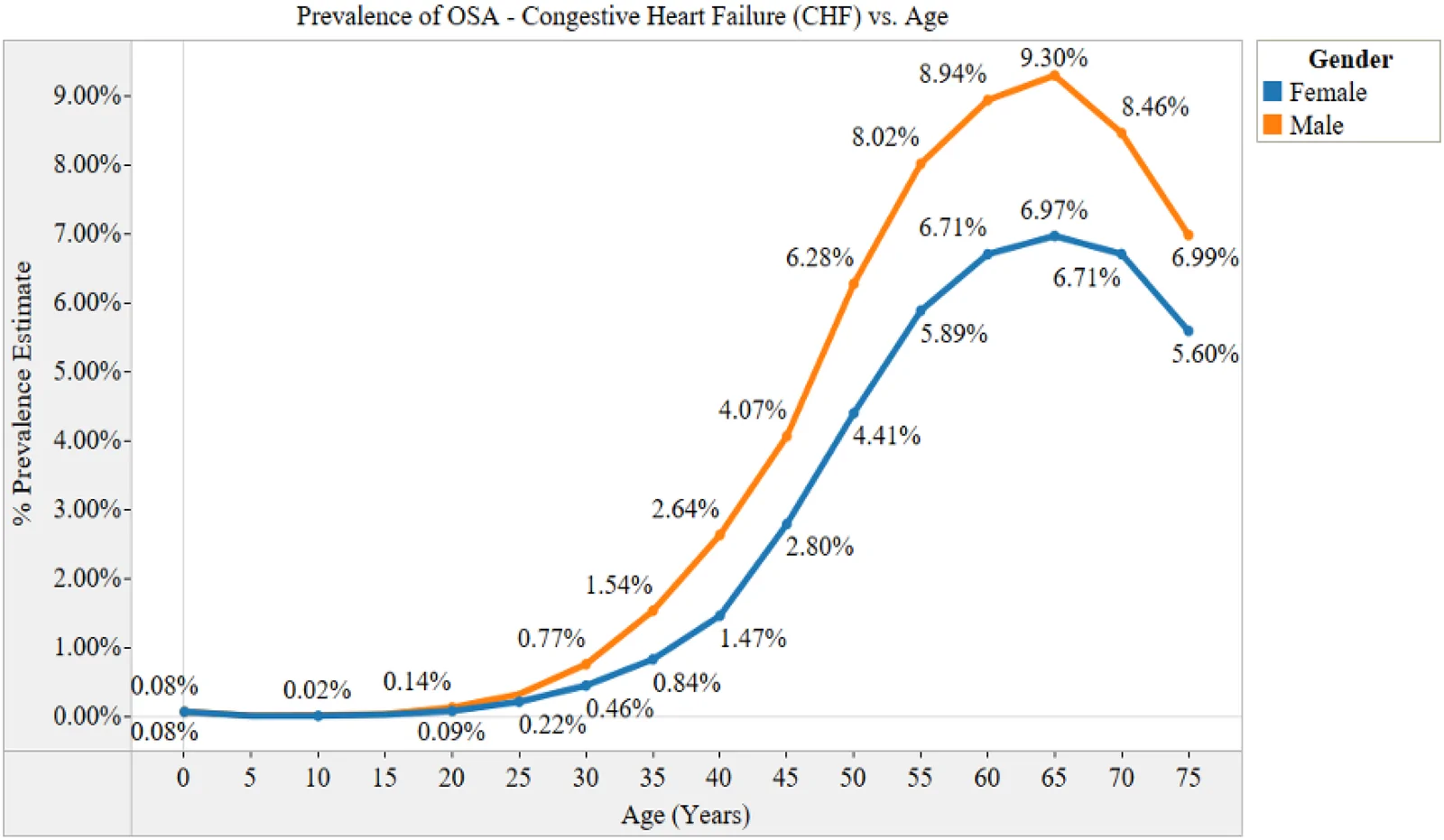
Prevalence estimate (%) – Congestive heart failure.

**Fig 13:**
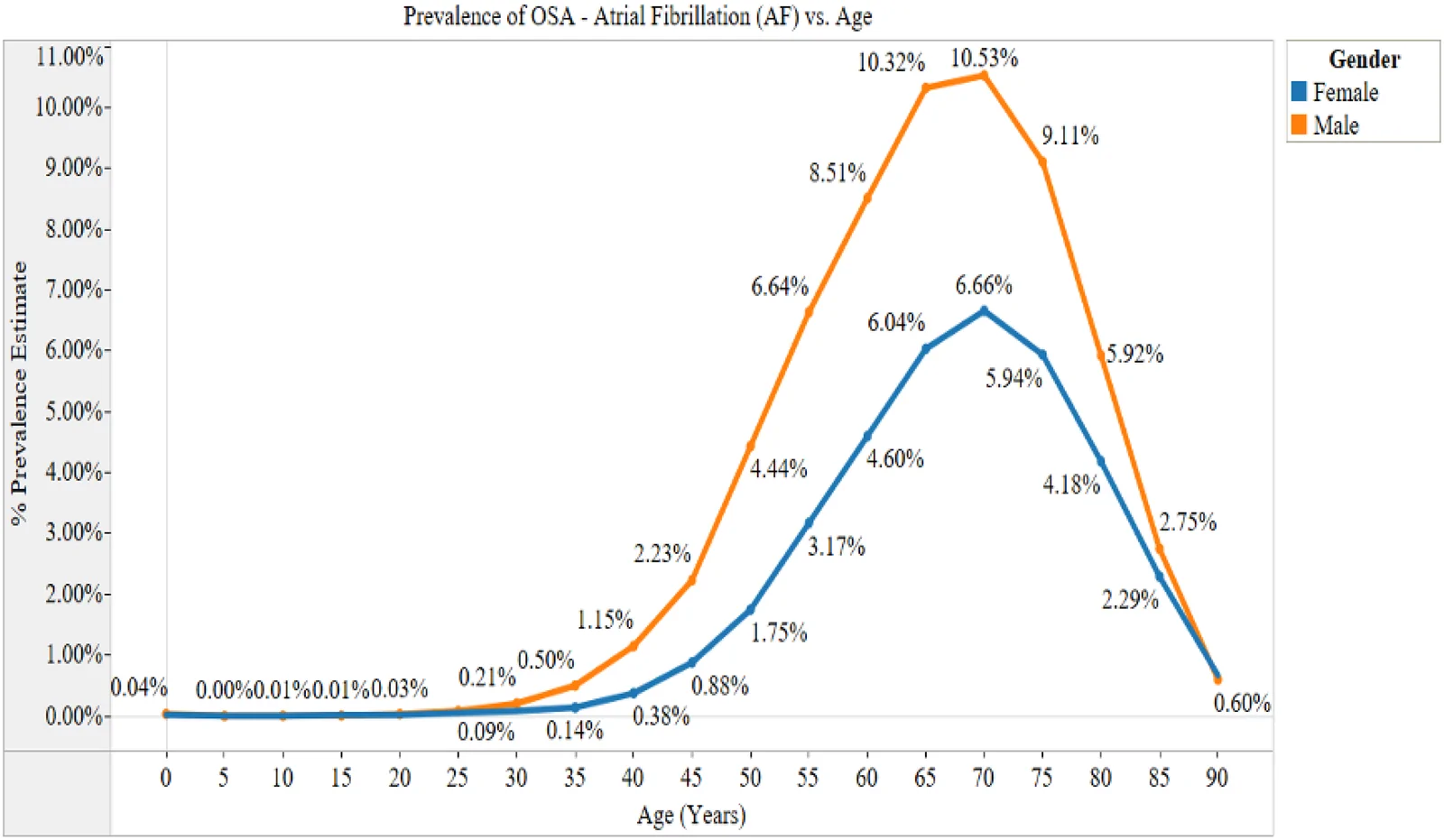
Prevalence estimate (%) – Atrial fibrillation.

Similarly, stroke and vascular disease prevalence peaked at 65 years of age for males. Stroke/TIA prevalence rates among OSA patients were 8.23% (n = 32,745) in males and 6.51% (n = 24,478) in females at age 60 (Fig 14). Vascular disease prevalence among OSA patients showed gender-specific peak ages: males at age 65 (10.48%; n = 32,651) and females at age 60 (5.62%; n = 13,714) (Fig 15). Table 8 summarizes the peak prevalence by comorbidity and gender.

**Table 8:** Peak prevalence rates and ages for comorbidities among OSA patients.

Comorbidity	Male			Female			Gender Difference
	Peak Age	Prevalence Rate	Sample Size	Peak Age	Prevalence Rate	Sample Size	Percentage Points (pp)
OSA	55	10.43%	32,589	55	9.28%	22,645	1.15 (M > F)
Hypertension	55	7.37%	23,016	55	7.11%	17,340	0.26 (M > F)
Diabetes	60	7.91%	24,715	60	7.58%	18,495	0.33 (M > F)
CHF	65	9.30%	29,048	65	6.97%	16,998	2.33 (M > F)
Atrial Fibrillation	70	10.53%	32,901	70	6.66%	16,229	3.87 (M > F)
Stroke/TIA	65	8.23%	32,745	60	6.51%	24,478	1.72 (M > F)
Vascular Disease	65	10.48%	32,651	60	5.62%	13,714	4.86 (M > F)

**Fig 14:**
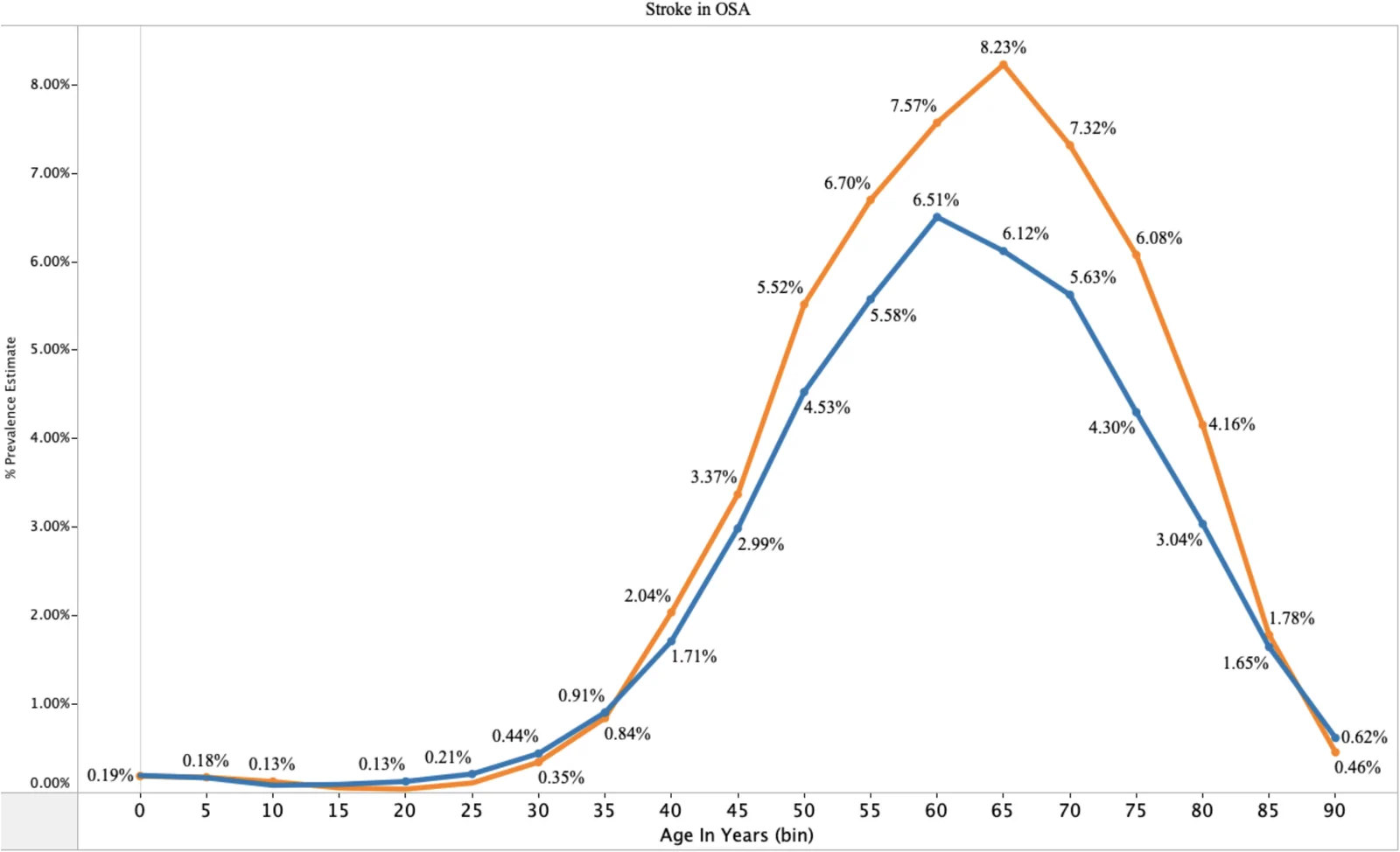
Prevalence estimate (%) – Stroke.

**Fig 15:**
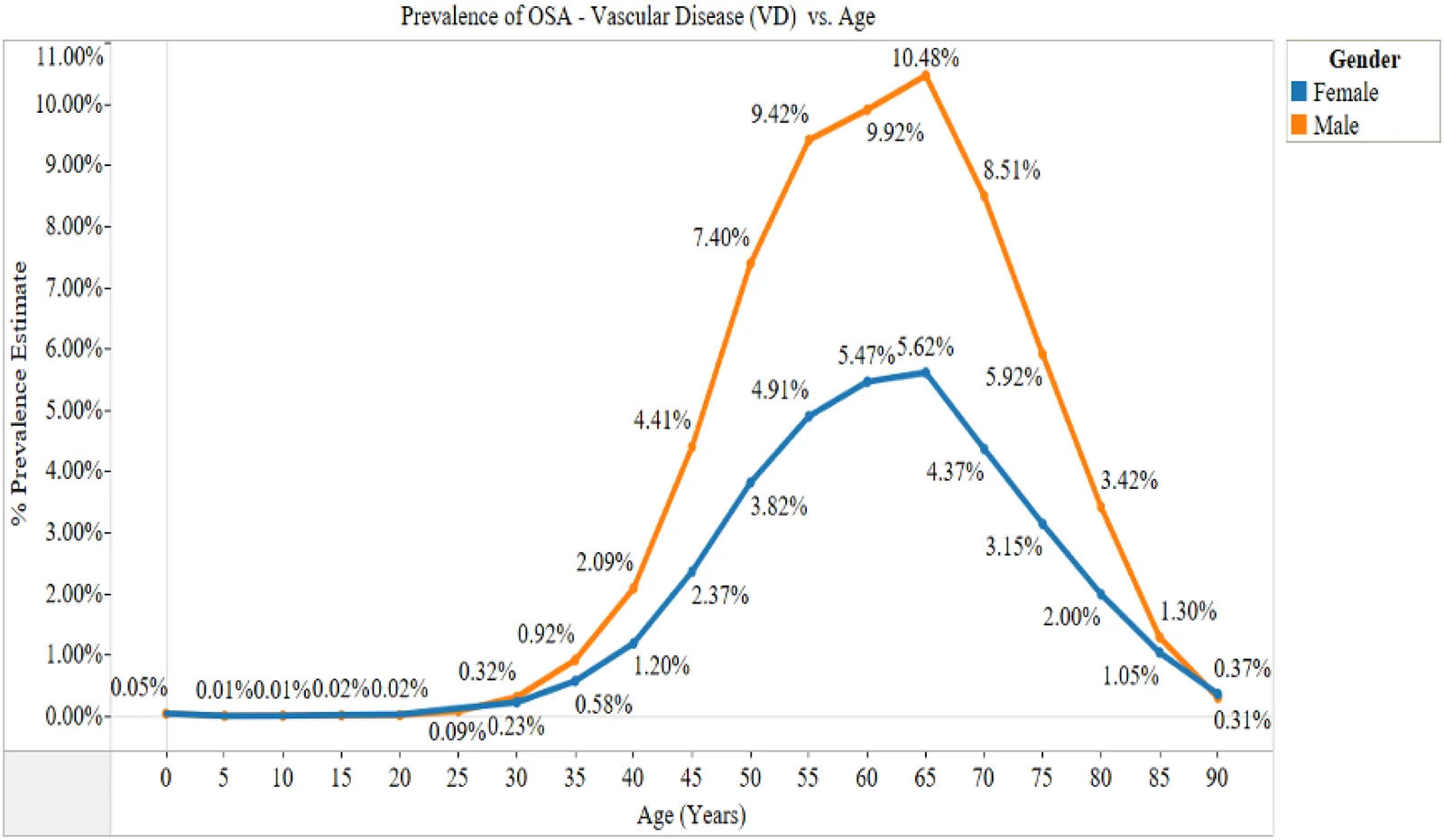
Prevalence estimate (%) – Vascular disease.

The patients with OSA demonstrated higher cardiovascular comorbidities in older age groups and represent cross-sectional age-specific prevalence estimates, not temporal progression. Prevalence rates for cardiovascular comorbidities are highest, followed by OSA, with the lowest rates for metabolic comorbidities (diabetes, hypertension). Moreover, cardiovascular comorbidities show larger disparities compared to metabolic comorbidities, with higher prevalence rates in males.

### Prevalence of major diagnostic categories (MDCs) in OSA patients

The descriptive results on prevalence between the OSA and MDC (Major Diagnostic Category) groups revealed differential patterns and impacts across major organ systems (Fig 16). *“Disease & Disorders of Ear, Nose, Mouth & Throat”* are most prevalent (n ~ 150,000). While *“Disease & Disorders of the Kidney & Urinary Tract”* are the least prevalent (n ~ 60,000). The results suggest that prevalence is pronounced in ENT, cardiovascular, and musculoskeletal body systems, consistent with the pathophysiology of chronic intermittent hypoxia in OSA patients.

**Fig 16:**
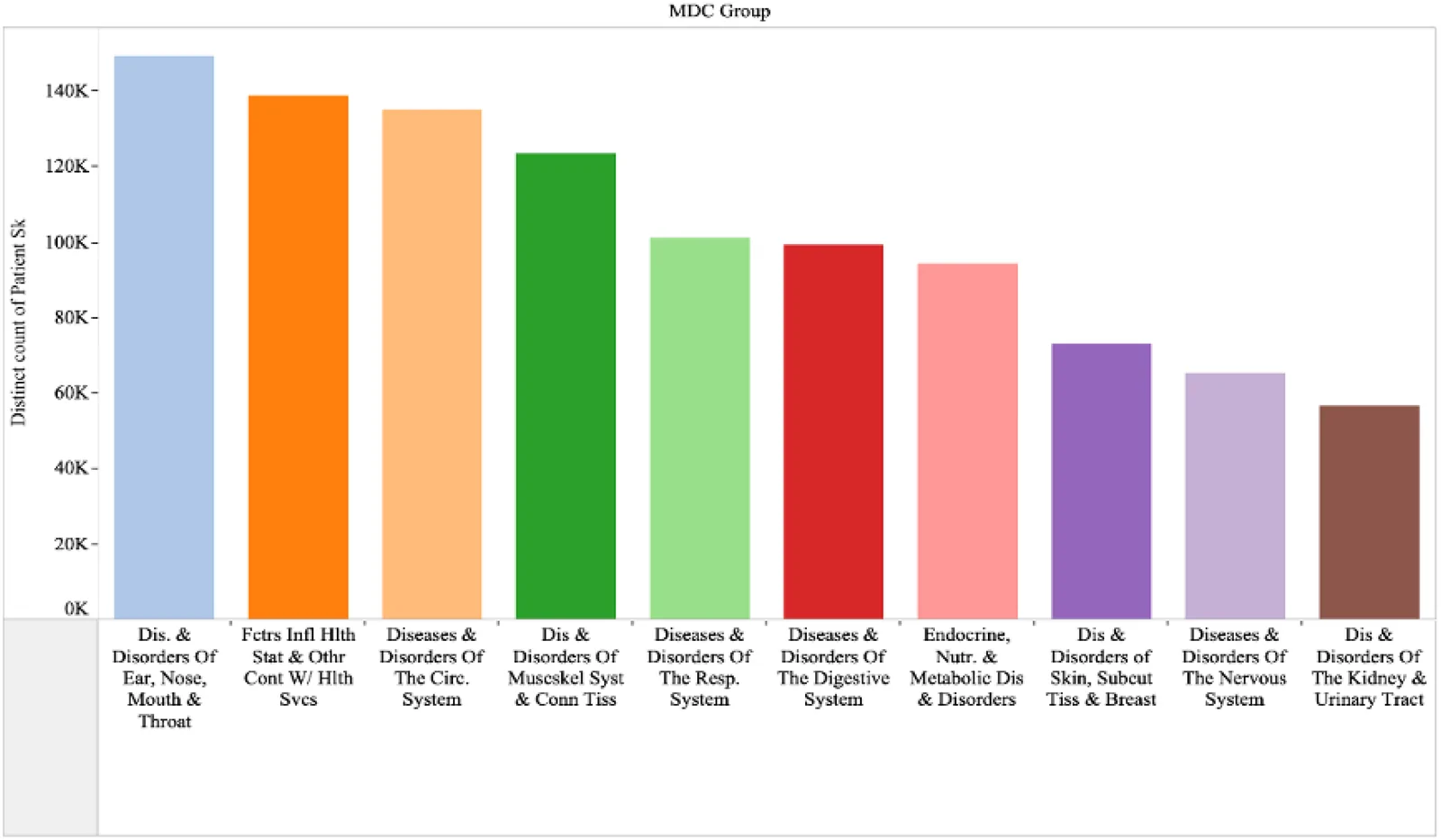
OSA population patterns by organ system (MDC).

Fig 17 shows the top 10 medical conditions among OSA patients, with stacked bar distributions by affected organ system (MDC group). Diabetes is the second most prevalent, followed by Esophageal reflux, showing that it exceeds respiratory and cardiovascular conditions. Across the top 10 conditions, the “Circulatory System” organ group consistently has a higher prevalence rate, suggesting the impact of OSA on the “Circulatory System” independent of the secondary comorbidities (Diabetes to Obesity). These results are consistent with Table 8 and Fig 16, which show the impact of OSA on the ENT, Respiratory, Musculoskeletal, and Digestive body systems.

**Fig 17:**
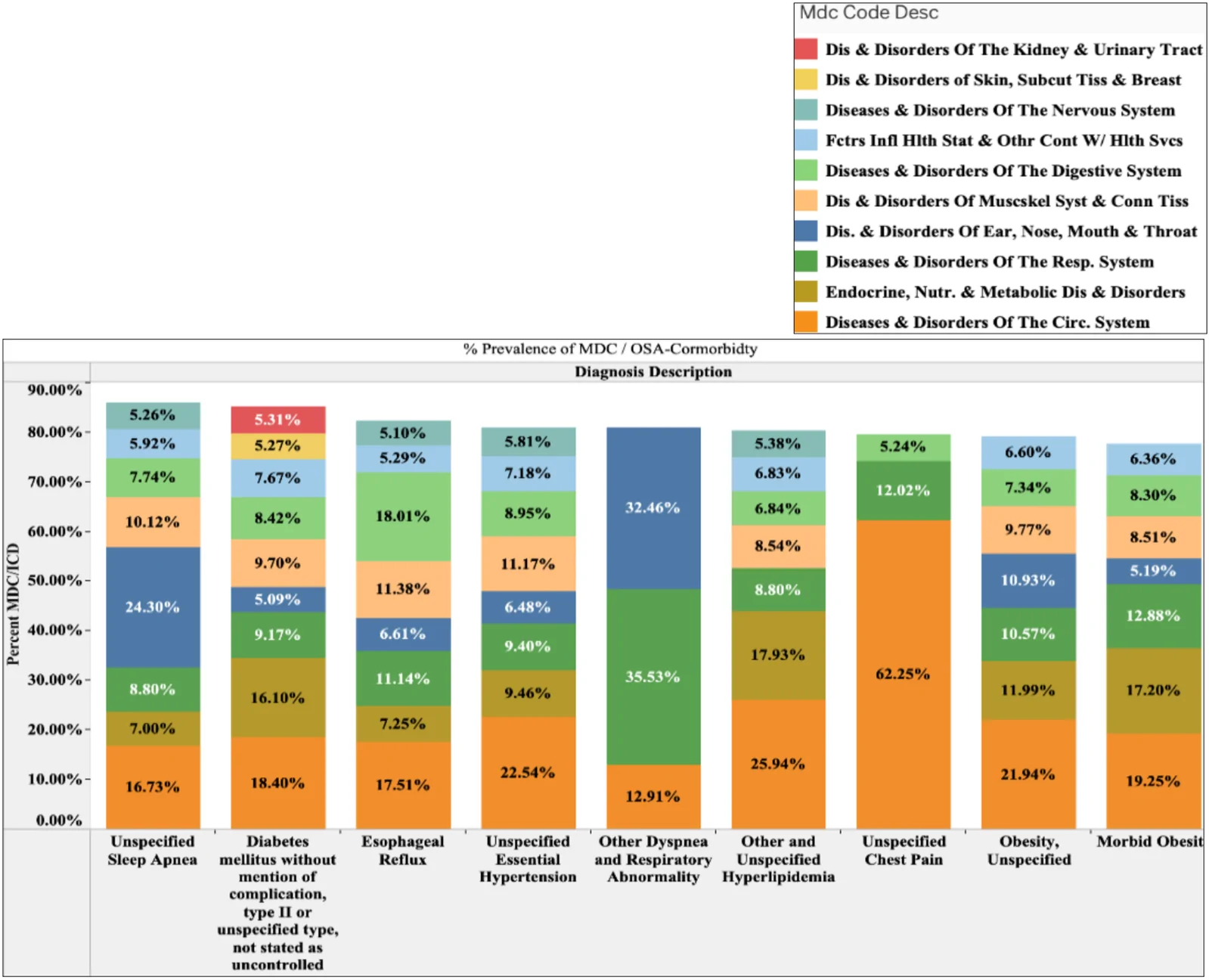
Percentage prevalence of OSA comorbidity and organ system (MDC).

## Discussion

The current study demonstrates the application of clinical research informatics for population-based epidemiological research of 556,474 OSA patients using a nationally representative EHR. The novel insight has implications with significance for stroke risk stratification and clinical screening for OSA. The primary findings and implications of the study are:

1. Prevalence of OSA was highest in the 55-year age group for both sexes (m = 10.43%, f = 9.28%), whereas the highest cross-sectional prevalence of cardiovascular comorbidities was observed in older age groups (65–70 years) (Fig 9, Table 8). While minor sex-specific differences were observed in age-stratified prevalence curves for vascular disease, highest prevalence estimates of stroke/TIA for both men and women indicate that the impact is similar for both genders.
2. Overall OSA prevalence was similar between sexes, but prevalence estimates of cardiovascular comorbidities were higher among males (Table 8). These findings show sex-specific differences. However, the differences do not represent risk progression. The reason for emphasizing prevalence progression is to show subgroups prevalence differences as evidence for comprehensive health management.
3. Although medication use and outcomes were not evaluated in the present study, identifying demographic subgroups with higher OSA prevalence may help prioritize screening efforts, and treatment of OSA and associated cardiovascular/metabolic comorbidities. Earlier identification of OSA, specifically in the younger female population, could reduce reliance on anti-thrombotic therapies such as aspirin. Aspirin is inferior to anti-coagulation therapies for stroke prevention in atrial fibrillation. Clinical guidelines discourage aspirin use to reduce side effects such as gastrointestinal and intracranial bleeding in adults with cardio-metabolic risk factors [38]. The above interpretations are hypothesis-generating and require validation in future studies.
4. Although this study does not evaluate stroke outcomes or incremental risk prediction, the descriptive prevalence patterns observed among ${\text{CHA}}_{2}{\text{DS}}_{2}-\text{VASc}$ components in OSA patients support future hypothesis-driven research to evaluate whether OSA provides independent or additive impact in longitudinal cohorts.
5. Lastly, MDC group analysis shows musculoskeletal system impact, highlighting OSA’s impact beyond cardiovascular and metabolic involvement. To the best of our knowledge, the broader impact of OSA based on organ systems (MDC) provides new clinical insights and has not been previously reported in the literature.

## Conclusion

This study is the first of a two-part study to demonstrate that descriptive clinical informatics can produce Level III evidence for EBM with precision, in a timely and cost-effective manner. The study demonstrates how future studies can be refined to develop higher-level evidence (Levels I or II).

The EHR data inherit temporal information to estimate longitudinal prevalence and extract causality, a drawback of traditional cross-sectional studies due to the lack of data on disease progression [31]. Cohort-based designs with repeated individual-level observations would be necessary to get accurate longitudinal inference and causal estimation. Figs 9–15 show the prevalence of all patient populations from newborns to patients aged 90 years. The results reflect the population’s prevalence and progression for both genders across all ages. Consequently, CRI with EHRs can bring a paradigm shift in epidemiological clinical research for other disease epidemics, such as Cancer, COPD, and Opioid addiction.

The currently used stroke risk stratification scheme, ${\text{CHA}}_{2}{\text{DS}}_{2}-\text{VASc}$, does not account for potential interactions among obstructive sleep apnea (OSA), stroke risk comorbidities, age, and gender. This absence of clinical guidelines limited cardiologists and sleep specialists at Oklahoma State University Medical Center in effective management of stroke risk in patients with atrial fibrillation (AF). We believe cardiologists have similar experiences at other healthcare operations. The study findings provide descriptive evidence of OSA prevalence stratified by age, sex, and associated stroke-risk comorbidities. Although the current study does not assess stroke risk prediction, the findings offer insight for evaluating the potential additive impact of OSA beyond established ${\text{CHA}}_{2}{\text{DS}}_{2}-\text{VASc}$ components.

### Study limitations

The current study does have its limitations. First, the cross-sectional design does not permit inference regarding temporal ordering, disease progression, or causality at the individual level. Observed age-specific differences reflect population-level prevalence patterns and may be influenced by cohort effects, diagnostic practices, or healthcare-seeking behavior. Second, prevalence estimates depend on documented diagnoses within the EHR; therefore, undiagnosed OSA or comorbid conditions may lead to underestimation of disease burden. Third, important clinical variables such as OSA severity, treatment adherence, medication use, obesity status, smoking history, family history, and socioeconomic factors were not available for analysis and may influence observed prevalence patterns. Finally, because the study population was derived from a single EHR repository, findings may not be fully generalizable to all healthcare settings or populations.

### Future directions

Future studies should explore predictive/prescriptive analytics to investigate the impact of OSA on stroke risk in AF patients and evaluate the stroke risk scheme (${\text{CHA}}_{2}{\text{DS}}_{2}-\text{VASc}$ score). Predictive models (logistic regression, decision tree) shall provide insight into relative risk among stroke risk disease and clinical understanding to explore possible causal mechanisms.

## Supporting information

S1 File Highlights. (PDF)
