# Supplementary material for: Age- and sex-stratified prevalence of obstructive sleep apnea and stroke risk comorbidities: A large cross-sectional EHR study
Source: PLoS One. 2026 Sep 30;21(9):e0357824. doi: 10.1371/journal.pone.0357824 (PMC13626327; doi:10.1371/journal.pone.0357824)
Supplement: Supplementary file 1 [file pone.0357824.s001.pdf]

Highlights

- Populations-Based epidemiological study of over 500,000 Obstructive Sleep Apnea (OSA) from a nationally representative EHR Data.
- Novel insight insights into the age- and gender-temporal patterns of OSA prevalence and associated stroke risk comorbidity.
- Establishes foundation with significance for stroke risk stratification in OSA patients.
- Demonstrates a novel and comprehensive application of clinical research informatics (CRI) for epidemiological research.
- First to show the prevalence connection between disease and major body organ system such as cardiovascular system.
