# Supplementary material for: Age- and sex-stratified prevalence of obstructive sleep apnea and stroke risk comorbidities: A large cross-sectional EHR study
Source: PLoS One. 2026 Sep 30;21(9):e0357824. doi: 10.1371/journal.pone.0357824 (PMC13626327; doi:10.1371/journal.pone.0357824)
Supplement: Supplementary file 2 [file pone.0357824.s002.pdf]

Age and gender specific Obstructive sleep apnea prevalence, and temporal lag to peak cardiovascular comorbidity a population based study.

The objective is different than the main title. It seems they are mixing cardiovascular comorbidity with stroke risk comorbidity.

But in results, they are again talking about cardiovascular co-morbidities.

In conclusion, I'm not sure why they use this word metabolic separating it from cardiovascular co morbidities .

There are so many studies out there, and there is no confusion that cardiovascular disease/atherosclerosis peaks about 5 to 10 years earlier in men than women. But stroke/TIA is more common in young woman like less than 45 but after 45 it's more common in men.
This paper is a little confusing to me as their objectives are not clear and their results are also not well defined.
I think they should clearly separate the cardiovascular risk and the stroke risk. They are mixing these two in their paper. I think it will create more confusion than broaden our knowledge.

In this paper, there are multiple locations where we have this error reference source not found. I think they should fix this.

Again, as I discussed in the review of literature, also they are talking about CHA2DS2 VASc score and less of cardiovascular co-morbidities.

In their results they are saying, despite relatively higher, clinical encounters and healthcare utilization among females OSA prevalence is higher in males than in females. I'm not sure what exactly they want to say here.

Giving a peak age like they're saying hypertension prevalence among OSA patients peaked at 55 years of both gender. I think it would have been better if they would have given a range of age not just one age. That would be clinically, more useful and reliable.

In their discussion, they are saying OSA prevalence is similar between genders while disparities exist in cardiovascular comorbidity with higher prevalence rates in males but before that they said OSA prevalence is also more in males.

Needs lot of corrections , better well defined objective and result discussion and

precise conclusions.
