# Supplementary material for: Age- and sex-stratified prevalence of obstructive sleep apnea and stroke risk comorbidities: A large cross-sectional EHR study
Source: PLoS One. 2026 Sep 30;21(9):e0357824. doi: 10.1371/journal.pone.0357824 (PMC13626327; doi:10.1371/journal.pone.0357824)
Supplement: Supplementary file 3 [file pone.0357824.s004.pdf]

Dear Academic Editor and Reviewers,

We sincerely thank the reviewers for their careful evaluation of our manuscript and for their constructive comments. We have revised the manuscript to address all concerns. Below we provide a detailed point-by-point response.

Academic Editor

Comment 1: Manuscript meeting style requirements.

Response: Thank you for this information. The manuscript and any associated files have been updated to meet PLOS One's requirements.

Comment 2: Manuscript should be in PLOS LaTeX format.

Response: The manuscript has been updated accordingly.

Comment 3: Funding information in Acknowledgments.

Response: Any statements regarding funding has been removed from the Acknowledgements section. The current Funding Statement should remain as is.

Comment 4: Restrictions to data sharing for this study.

Response: Thank you for the clarification regarding PLOS ONE's data availability requirements.

a) The data used in this study were obtained from the Cerner HealthFacts® database, which is a proprietary, third-party electronic health record (EHR) repository owned by Cerner Corporation. Although the dataset was provided to the investigators in de-identified form, there are legal restrictions imposed by Cerner Corporation that prohibit public redistribution of the data. The authors do not have the authority to make the underlying data publicly available. Researchers who meet the criteria for access to confidential data may request access directly from Cerner through an institutional agreement.
b) Given the legal and contractual restrictions described above, the authors are unable to deposit the dataset in a public repository or provide it as Supporting Information.

Comment 5: Separate caption for each figure.

Response: Captions have been included in the updated manuscript for each figure.

Comment 6: Figure references.

Response: All figures have been validated and referenced throughout the manuscript.

Reviewer #1

Comment 1: Title and objectives are confusing; cardiovascular comorbidities and stroke risk factors are used interchangeably. Hypertension appears separated from cardiovascular risk factors without clarity.

Response: We thank the reviewer for this comment. We have revised the title, abstract, and objectives to clearly distinguish cardiovascular comorbidities from stroke-risk components within the CHA_2DS_2-VASc framework. We also define analytic categories and justify the separation of metabolic (hypertension, diabetes) and cardiovascular comorbidities in the Introduction.

Comment 2: Multiple instances of "error reference source not found."

Response: All figure and table references have been corrected.

Reviewer #2

Comment 1: Cross-sectional design mislabeled; temporal and causal language overreached.

Response: We agree and have comprehensively revised the manuscript to eliminate causal or temporal interpretations. Terms such as "temporal lag" and "intervention window" have been removed or reframed. The cross-sectional nature and its limitations are now explicitly acknowledged in the Abstract, Methods, Results, Discussion, and Limitations sections.

Comment 2: Internal inconsistencies in peak prevalence values (Table 8 vs text).

Response: All prevalence estimates and peak age values have been re-validated. The Results, Abstract, and Discussion now consistently align with Table 8, and we clarified interpretations of sex-specific age differences.

Comment 3: Claim that OSA should be added to CHA_2DS_2-VASc is unsupported.

Response: We appreciate this important clarification. We have reframed all language implying modification or revision of CHA_2DS_2-VASc. The manuscript now explicitly states that no stroke

outcomes or predictive modeling were performed, and that findings are descriptive and hypothesis-generating, intended to inform future longitudinal evaluation rather than guideline changes.

Comment 4: Gender-specific intervention claims are not supported.

Response: We have removed prescriptive language regarding “early intervention for females.” Sex-specific findings are now presented strictly as population-level prevalence patterns. The Discussion reframes potential implications as hypotheses related to downstream management considerations, explicitly noting that medication use, and outcomes were not assessed.

We believe these revisions substantially improve conceptual clarity, methodological rigor, and interpretive accuracy while preserving the study’s contribution as a large, population-based descriptive analysis. We are grateful for the reviewers’ guidance and hope the revised manuscript is now suitable for publication.

Sincerely,

Corresponding Author
