# Supplementary material for: Age- and sex-stratified prevalence of obstructive sleep apnea and stroke risk comorbidities: A large cross-sectional EHR study
Source: PLoS One. 2026 Sep 30;21(9):e0357824. doi: 10.1371/journal.pone.0357824 (PMC13626327; doi:10.1371/journal.pone.0357824)
Supplement: Supplementary file 4 [file pone.0357824.s005.pdf]

Dear Academic Editor and Reviewers,

We sincerely thank the reviewers for their careful evaluation of our manuscript and for their constructive comments. We have revised the manuscript to address all the concerns. Below, we provide a detailed point-by-point response.

Reviewer #1

Comment 1: There appear to be discordance between the study objectives and final conclusions.

Response 1: We revised the Conclusion to better align with the study objective and cross-sectional design. The revised text now emphasizes descriptive prevalence patterns and their implications for future longitudinal research rather than modification of the CHA_2DS_2-VASc scheme.

Comment 2: Figure 5 summarizes the patient selection and data extraction. I do not see figure 5 in the presented manuscript.

Response 2: We corrected the figure citation. Patient selection is summarized in Figure 4, while Figures 5–7 present demographic distributions.

Comment 3: It is noteworthy that the OSA prevalence peaks at birth and dips to its lowest at age 20.This statement appears factually inaccurate. Please provide reference is available.

Response 3: We agreed and removed the statement. The revised text now focuses on age-specific prevalence patterns within the study cohort.

Comment 4: OSA Prevalence by Age and Gender. This whole paragraph is confusing and should be rewritten and numbers should be checked again if total OSA pt included in the study are 556474.

Response 4: We rewrote the "OSA Prevalence by Age and Gender" and "OSA Comorbidity Prevalence by Age and Gender" sections for clarity. We now explicitly state that prevalence estimates were calculated within each sex-specific OSA population (males: n = 312,450; females: n = 244,024), not the overall cohort. All percentages, sample sizes, and prevalence estimates were rechecked and verified.

Comment 5: Limitations are not clearly defined.

Response 5: We expanded the Study Limitations section to more clearly describe the limitations of the cross-sectional design, potential confounding factors, undiagnosed conditions, and issues related to generalizability.

Comment 6: OSA screening may pause or at least reduce the cardiovascular and metabolic complications. Please explain little more under discussion.

Response 6: We expanded the Discussion to clarify that identifying demographic subgroups with higher OSA prevalence may help prioritize screening efforts and support earlier recognition and management of associated cardiovascular and metabolic comorbidities. We also note that these implications are hypothesis-generating and require validation in future studies.

We thank Reviewer #1 for the constructive comments and suggestions. We have carefully addressed each point and believe that the revised manuscript now fully addresses all reviewer concerns. We hope that the revisions meet the expectations of the reviewer and editor, and we respectfully submit the manuscript for further consideration for publication.

Sincerely,
Corresponding Author
