# Supplementary material for: Age- and sex-stratified prevalence of obstructive sleep apnea and stroke risk comorbidities: A large cross-sectional EHR study
Source: PLoS One. 2026 Sep 30;21(9):e0357824. doi: 10.1371/journal.pone.0357824 (PMC13626327; doi:10.1371/journal.pone.0357824)
Supplement: Peer review history

# Peer review: Decision Letter 0

**DOI:** 10.1371/journal.pone.0357824.r001

**Authors:** Jamal Akhtar

1 Apr 2026

Dear Dr. Agrawal,

Thank you for submitting your manuscript to PLOS ONE. After careful consideration, we feel that it has merit but does not fully meet PLOS ONE’s publication criteria as it currently stands. Therefore, we invite you to submit a revised version of the manuscript that addresses the points raised during the review process.

Please submit your revised manuscript by May 16 2026 11:59PM. If you will need more time than this to complete your revisions, please reply to this message or contact the journal office at plosone@plos.org. When you're ready to submit your revision, log on to https://www.editorialmanager.com/pone/ and select the 'Submissions Needing Revision' folder to locate your manuscript file.

- A letter that responds to each point raised by the academic editor and reviewer(s). You should upload this letter as a separate file labeled 'Response to Reviewers'.
- A marked-up copy of your manuscript that highlights changes made to the original version. You should upload this as a separate file labeled 'Revised Manuscript with Track Changes'.
- An unmarked version of your revised paper without tracked changes. You should upload this as a separate file labeled 'Manuscript'.

If you would like to make changes to your financial disclosure, please include your updated statement in your cover letter. Guidelines for resubmitting your figure files are available below the reviewer comments at the end of this letter.

If applicable, we recommend that you deposit your laboratory protocols in protocols.io to enhance the reproducibility of your results. Protocols.io assigns your protocol its own identifier (DOI) so that it can be cited independently in the future. For instructions see: https://journals.plos.org/plosone/s/submission-guidelines#loc-laboratory-protocols. Additionally, PLOS ONE offers an option for publishing peer-reviewed Lab Protocol articles, which describe protocols hosted on protocols.io. Read more information on sharing protocols at https://plos.org/protocols?utm_medium=editorial-email&utm_source=authorletters&utm_campaign=protocols.

As the corresponding author, your ORCID iD is verified in the submission system and will appear in the published article. PLOS supports the use of ORCID, and we encourage all coauthors to register for an ORCID iD and use it as well. Please encourage your coauthors to verify their ORCID iD within the submission system before final acceptance, as unverified ORCID iDs will not appear in the published article. *Only*  the individual author can complete the verification step; PLOS staff *cannot*  verify ORCID iDs on behalf of authors.

We look forward to receiving your revised manuscript.

Kind regards,

Jamal Akhtar

Academic Editor

PLOS One

Journal Requirements:

When submitting your revision, we need you to address these additional requirements.

1. Please ensure that your manuscript meets PLOS ONE's style requirements, including those for file naming. The PLOS ONE style templates can be found at

https://journals.plos.org/plosone/s/file?id=wjVg/PLOSOne_formatting_sample_main_body.pdf and

https://journals.plos.org/plosone/s/file?id=ba62/PLOSOne_formatting_sample_title_authors_affiliations.pdf

2. Please update your submission to use the PLOS LaTeX template. The template and more information on our requirements for LaTeX submissions can be found at http://journals.plos.org/plosone/s/latex.

3. Thank you for stating the following in the Acknowledgments Section of your manuscript:

“The authors received no financial support to complete the study.”

We note that you have provided funding information that is not currently declared in your Funding Statement. However, funding information should not appear in the Acknowledgments section or other areas of your manuscript. We will only publish funding information present in the Funding Statement section of the online submission form.

Please remove any funding-related text from the manuscript and let us know how you would like to update your Funding Statement. Currently, your Funding Statement reads as follows:

“The author(s) received no specific funding for this work.”

Please include your amended statements within your cover letter; we will change the online submission form on your behalf.

4. We note that you have indicated that there are restrictions to data sharing for this study. PLOS only allows data to be available upon request if there are legal or ethical restrictions on sharing data publicly. For more information on unacceptable data access restrictions, please see http://journals.plos.org/plosone/s/data-availability#loc-unacceptable-data-access-restrictions.

Before we proceed with your manuscript, please address the following prompts:

a) If there are ethical or legal restrictions on sharing a de-identified data set, please explain them in detail (e.g., data contain potentially identifying or sensitive patient information, data are owned by a third-party organization, etc.) and who has imposed them (e.g., a Research Ethics Committee or Institutional Review Board, etc.). Please also provide contact information for a data access committee, ethics committee, or other institutional body to which data requests may be sent.

b) If there are no restrictions, please upload the minimal anonymized data set necessary to replicate your study findings to a stable, public repository and provide us with the relevant URLs, DOIs, or accession numbers. For a list of recommended repositories, please see

https://journals.plos.org/plosone/s/recommended-repositories. You also have the option of uploading the data as Supporting Information files, but we would recommend depositing data directly to a data repository if possible.

We will update your Data Availability statement on your behalf to reflect the information you provide.

5. Please include a separate caption for each figure in your manuscript.

6. Please ensure that you refer to Figures 1 - 4 and 6 - 16 in your text as, if accepted, production will need this reference to link the reader to the figure.

7. If the reviewer comments include a recommendation to cite specific previously published works, please review and evaluate these publications to determine whether they are relevant and should be cited. There is no requirement to cite these works unless the editor has indicated otherwise.

[Note: HTML markup is below. Please do not edit.]

Reviewers' comments:

Reviewer's Responses to Questions

**Comments to the Author**

1. Is the manuscript technically sound, and do the data support the conclusions?

Reviewer #1: Partly

Reviewer #2: Partly

**********

2. Has the statistical analysis been performed appropriately and rigorously?

Reviewer #1: I Don't Know

Reviewer #2: Yes

**********

3. Have the authors made all data underlying the findings in their manuscript fully available?

The PLOS Data policy

Reviewer #1: Yes

Reviewer #2: Yes

**********

4. Is the manuscript presented in an intelligible fashion and written in standard English?

Reviewer #1: No

Reviewer #2: Yes

**********

Reviewer #1: The title and objective are different and confusing. Cardiovascular co-morbidities, and stroke Risk factors are not the same. It seems at different places they have useed this two interchangeably. the way they have written, it seems they have separated hypertension from cardiovascular risk factors. At multiple places showing up error reference source not found. I don't see anything very relevant for clinical practice that i can take from this paper. more clarity in defining objectives , discussion , conclusions and results needed.

Reviewer #2: Several major methodological, analytical, and interpretive concerns must be addressed before this manuscript is suitable for publication.

1. Study design mislabeled and causal language overreached

The authors repeatedly use temporal and causal language (e.g., "temporal lag," "critical intervention window," "OSA may cause cardiovascular complications") despite this being a cross-sectional prevalence study. The age-stratified cross-sectional design cannot establish temporal sequence or causality. The "temporal lag" observed between OSA and cardiovascular comorbidity prevalence peaks is an artifact of cross-sectional age-stratified data and does not represent a longitudinal trajectory for individual patients. The authors must clearly and consistently acknowledge this limitation throughout the manuscript, including the title and abstract, and rephrase all causal or temporal language accordingly.

2. Peak prevalence figures appear internally inconsistent

In Table 8, AF peak prevalence in males is reported as 10.53 at age 70, yet in the text, AF prevalence rates are also reported as 10.4848 in males at age 60. Similarly, Vascular Disease sample sizes for males (n=32,651) and females (n=13,714) at different peak ages are identical to Stroke/TIA figures, suggesting possible copy-paste errors. The authors must verify and reconcile all figures in Table 8 with those reported in the text and figures.

3. The claim that OSA should be added to CHA2DS2-VASc is not supported by the data

The study demonstrates prevalence patterns of CHA2DS2-VASc comorbidities in OSA patients but does not test whether OSA independently predicts stroke risk beyond existing CHA2DS2-VASc factors, nor does it provide hazard ratios, odds ratios, or any inferential statistics comparing OSA patients to non-OSA controls. The conclusion that CHA2DS2-VASc should be revised to include OSA is not supported by the descriptive data presented. This conclusion should be substantially toned down or reframed as a hypothesis for future research.

4. Gender-specific findings require clarification

The abstract and conclusion state that "early intervention for females" is warranted and that stroke/TIA and vascular disease peak at different ages by gender (females age 60, males age 65). However, Table 8 shows the same peak age (65) for Stroke/TIA in both genders, which contradicts the text. These discrepancies must be resolved. Additionally, the rationale for "early intervention in females" based on prevalence data alone is not sufficiently supported.

5. Several broken figure and table references throughout the manuscript

The manuscript contains numerous instances of "Error! Reference source not found." where figures and supplementary tables should be cited. These must all be corrected before submission.

6. Reference 27 appears to be incorrectly cited

Reference 27 (Garvey et al., 2019) is cited in the context of CPAP adherence but describes a study about queer and trans students navigating college contexts, appears to be a citation error.

OVERALL RECOMMENDATION

This study has potential value as a large-scale descriptive epidemiological analysis of OSA comorbidity patterns. However, the causal overreach in the interpretation, internal inconsistencies in reported data, broken figure references, and unsupported conclusions regarding CHA2DS2-VASc revision require major revision before this manuscript can be considered for publication.

**********

what does this mean?). If published, this will include your full peer review and any attached files.

If you choose “no”, your identity will remain anonymous but your review may still be made public.

**Do you want your identity to be public for this peer review?**  For information about this choice, including consent withdrawal, please see our Privacy Policy

Reviewer #1: **Yes:** Devendra Tripathi

Reviewer #2: No

**********

[NOTE: If reviewer comments were submitted as an attachment file, they will be attached to this email and accessible via the submission site. Please log into your account, locate the manuscript record, and check for the action link "View Attachments". If this link does not appear, there are no attachment files.]

To ensure your figures meet our technical requirements, please review our figure guidelines: https://journals.plos.org/plosone/s/figures

You may also use PLOS’s free figure tool, NAAS, to help you prepare publication quality figures: https://journals.plos.org/plosone/s/figures#loc-tools-for-figure-preparation.

NAAS will assess whether your figures meet our technical requirements by comparing each figure against our figure specifications.

Attachment Submitted filename: renamed_eb2a1.pdf

# Author comment: Author response to Decision Letter 1

**DOI:** 10.1371/journal.pone.0357824.r002

25 May 2026

Reviewer #1

Comment 1: Title and objectives are confusing; cardiovascular comorbidities and stroke risk factors are used interchangeably. Hypertension appears separated from cardiovascular risk factors without clarity.

Response: We thank the reviewer for this comment. We have revised the title, abstract, and objectives to clearly distinguish cardiovascular comorbidities from stroke risk components within the CHA₂DS₂ VASc framework. We also define analytic categories and justify the separation of metabolic (hypertension, diabetes) and cardiovascular comorbidities in the Introduction.

Comment 2: Multiple instances of “error reference source not found.”

Response: All figure and table references have been corrected.

Reviewer #2

Comment 1: Cross sectional design mislabeled; temporal and causal language overreached.

Response: We agree and have comprehensively revised the manuscript to eliminate causal or temporal interpretations. Terms such as “temporal lag” and “intervention window” have been removed or reframed. The cross sectional nature and its limitations are now explicitly acknowledged in the Abstract, Methods, Results, Discussion, and Limitations sections.

Comment 2: Internal inconsistencies in peak prevalence values (Table 8 vs text).

Response: All prevalence estimates and peak age values have been re validated. The Results, Abstract, and Discussion now consistently align with Table 8, and we clarified interpretations of sex specific age differences.

Comment 3: Claim that OSA should be added to CHA₂DS₂ VASc is unsupported.

Response: We appreciate this important clarification. We have reframed all language implying modification or revision of CHA₂DS₂ VASc. The manuscript now explicitly states that no stroke outcomes or predictive modeling were performed, and that findings are descriptive and hypothesis generating, intended to inform future longitudinal evaluation rather than guideline changes.

Comment 4: Gender specific intervention claims are not supported.

Response: We have removed prescriptive language regarding “early intervention for females.” Sex specific findings are now presented strictly as population level prevalence patterns. The Discussion reframes potential implications as hypotheses related to downstream management considerations, explicitly noting that medication use, and outcomes were not assessed.

Attachment Submitted filename: Response to Reviewers.pdf

# Peer review: Decision Letter 1

**DOI:** 10.1371/journal.pone.0357824.r003

**Authors:** Omid Beiki

11 Aug 2026

Dear Dr. Agrawal,

Thank you for submitting your manuscript to PLOS ONE. After careful consideration, we feel that it has merit but does not fully meet PLOS ONE’s publication criteria as it currently stands. Therefore, we invite you to submit a revised version of the manuscript that addresses the points raised during the review process.

Please submit your revised manuscript by Sep 25 2026 11:59PM. If you will need more time than this to complete your revisions, please reply to this message or contact the journal office at plosone@plos.org. When you're ready to submit your revision, log on to https://www.editorialmanager.com/pone/ and select the 'Submissions Needing Revision' folder to locate your manuscript file.

- A letter that responds to each point raised by the academic editor and reviewer(s). You should upload this letter as a separate file labeled 'Response to Reviewers'.
- A marked-up copy of your manuscript that highlights changes made to the original version. You should upload this as a separate file labeled 'Revised Manuscript with Track Changes'.
- An unmarked version of your revised paper without tracked changes. You should upload this as a separate file labeled 'Manuscript'.

If you would like to make changes to your financial disclosure, please include your updated statement in your cover letter. Guidelines for resubmitting your figure files are available below the reviewer comments at the end of this letter.

If applicable, we recommend that you deposit your laboratory protocols in protocols.io to enhance the reproducibility of your results. Protocols.io assigns your protocol its own identifier (DOI) so that it can be cited independently in the future. For instructions see: https://journals.plos.org/plosone/s/submission-guidelines#loc-laboratory-protocols. Additionally, PLOS ONE offers an option for publishing peer-reviewed Lab Protocol articles, which describe protocols hosted on protocols.io. Read more information on sharing protocols at https://plos.org/protocols?utm_medium=editorial-email&utm_source=authorletters&utm_campaign=protocols.

As the corresponding author, your ORCID iD is verified in the submission system and will appear in the published article. PLOS supports the use of ORCID, and we encourage all coauthors to register for an ORCID iD and use it as well. Please encourage your coauthors to verify their ORCID iD within the submission system before final acceptance, as unverified ORCID iDs will not appear in the published article. *Only*  the individual author can complete the verification step; PLOS staff *cannot*  verify ORCID iDs on behalf of authors.

We look forward to receiving your revised manuscript.

Kind regards,

Omid Beiki, MD, PhD, MSc

Academic Editor

PLOS One

Journal Requirements:

If the reviewer comments include a recommendation to cite specific previously published works, please review and evaluate these publications to determine whether they are relevant and should be cited. There is no requirement to cite these works unless the editor has indicated otherwise.

[Note: HTML markup is below. Please do not edit.]

Reviewers' comments:

Reviewer's Responses to Questions

**Comments to the Author**

Reviewer #1: All comments have been addressed

Reviewer #2: All comments have been addressed

**********

2. Is the manuscript technically sound, and do the data support the conclusions?

Reviewer #1: No

Reviewer #2: Yes

**********

3. Has the statistical analysis been performed appropriately and rigorously?

Reviewer #1: No

Reviewer #2: Yes

**********

4. Have the authors made all data underlying the findings in their manuscript fully available?

The PLOS Data policy

Reviewer #1: No

Reviewer #2: Yes

**********

5. Is the manuscript presented in an intelligible fashion and written in standard English?

Reviewer #1: Yes

Reviewer #2: Yes

**********

Reviewer #1: 1. there appear to be discordance between the study objectives and final conclusions.

2. Figure 5 summarizes the patient selection and data extraction. I do not see figure 5 in the presented manuscript.

3.It is noteworthy that the OSA prevalence peaks at birth and dips to its lowest at age 20.This statement appears factually inaccurate. please provide reference is available.

4.OSA Prevalence by Age and Gender. this whole paragraph is confusing and should be rewritten and numbers should be checked again if total OSA pt included in the study are 556474.

5.limitations are not clearly defined.

6OSA screening may pause or at least reduce the cardiovascular and metabolic complications. please explain little more under discussion.

Reviewer #2: All comments have been adequately addressed. no further comments at this time, would recommend proceeding with publication

**********

what does this mean?). If published, this will include your full peer review and any attached files.

If you choose “no”, your identity will remain anonymous but your review may still be made public.

**Do you want your identity to be public for this peer review?**  For information about this choice, including consent withdrawal, please see our Privacy Policy

Reviewer #1: **Yes:** Devendra Tripathi

Reviewer #2: No

**********

[NOTE: If reviewer comments were submitted as an attachment file, they will be attached to this email and accessible via the submission site. Please log into your account, locate the manuscript record, and check for the action link "View Attachments". If this link does not appear, there are no attachment files.]

To ensure your figures meet our technical requirements, please review our figure guidelines: https://journals.plos.org/plosone/s/figures

You may also use PLOS’s free figure tool, NAAS, to help you prepare publication quality figures: https://journals.plos.org/plosone/s/figures#loc-tools-for-figure-preparation.

NAAS will assess whether your figures meet our technical requirements by comparing each figure against our figure specifications.

# Author comment: Author response to Decision Letter 2

**DOI:** 10.1371/journal.pone.0357824.r004

18 Aug 2026

Dear Academic Editor and Reviewers,

We sincerely thank the reviewers for their careful evaluation of our manuscript and for their constructive comments. We have revised the manuscript to address all the concerns. Below, we provide a detailed point-by-point response.

Reviewer #1

Comment 1: There appear to be discordance between the study objectives and final conclusions.

Response 1: We revised the Conclusion to better align with the study objective and cross-sectional design. The revised text now emphasizes descriptive prevalence patterns and their implications for future longitudinal research rather than modification of the CHA₂DS₂-VASc scheme.

Comment 2: Figure 5 summarizes the patient selection and data extraction. I do not see figure 5 in the presented manuscript.

Response 2: We corrected the figure citation. Patient selection is summarized in Figure 4, while Figures 5–7 present demographic distributions.

Comment 3: It is noteworthy that the OSA prevalence peaks at birth and dips to its lowest at age 20.This statement appears factually inaccurate. Please provide reference is available.

Response 3: We agreed and removed the statement. The revised text now focuses on age-specific prevalence patterns within the study cohort.

Comment 4: OSA Prevalence by Age and Gender. This whole paragraph is confusing and should be rewritten and numbers should be checked again if total OSA pt included in the study are 556474.

Response 4: We rewrote the “OSA Prevalence by Age and Gender” and “OSA Comorbidity Prevalence by Age and Gender” sections for clarity. We now explicitly state that prevalence estimates were calculated within each sex-specific OSA population (males: n = 312,450; females: n = 244,024), not the overall cohort. All percentages, sample sizes, and prevalence estimates were rechecked and verified.

Comment 5: Limitations are not clearly defined.

Response 5: We expanded the Study Limitations section to more clearly describe the limitations of the cross-sectional design, potential confounding factors, undiagnosed conditions, and issues related to generalizability.

Comment 6: OSA screening may pause or at least reduce the cardiovascular and metabolic complications. Please explain little more under discussion.

Response 6: We expanded the Discussion to clarify that identifying demographic subgroups with higher OSA prevalence may help prioritize screening efforts and support earlier recognition and management of associated cardiovascular and metabolic comorbidities. We also note that these implications are hypothesis-generating and require validation in future studies.

We thank Reviewer #1 for the constructive comments and suggestions. We have carefully addressed each point and believe that the revised manuscript now fully addresses all reviewer concerns. We hope that the revisions meet the expectations of the reviewer and editor, and we respectfully submit the manuscript for further consideration for publication.

Sincerely,

Corresponding Author

Attachment Submitted filename: Response_to_Reviewers_auresp_2.pdf

# Peer review: Decision Letter 2

**DOI:** 10.1371/journal.pone.0357824.r005

**Authors:** Omid Beiki

23 Aug 2026

Age - and sex - stratified prevalence of obstructive sleep apnea and stroke risk comorbidities: A large cross-sectional EHR study

PONE-D-26-00877R2

Dear Dr. Agrawal,

We’re pleased to inform you that your manuscript has been judged scientifically suitable for publication and will be formally accepted for publication once it meets all outstanding technical requirements.

Within one week, you’ll receive an e-mail detailing the required amendments. When these have been addressed, you’ll receive a formal acceptance letter and your manuscript will be scheduled for publication.

An invoice will be generated when your article is formally accepted. Please note, if your institution has a publishing partnership with PLOS and your article meets the relevant criteria, all or part of your publication costs will be covered. Please make sure your user information is up-to-date by logging into Editorial Manager at Editorial Manager® and clicking the ‘Update My Information' link at the top of the page. For questions related to billing, please contact billing support.

If your institution or institutions have a press office, please notify them about your upcoming paper to help maximize its impact. If they’ll be preparing press materials, please inform our press team as soon as possible -- no later than 48 hours after receiving the formal acceptance. Your manuscript will remain under strict press embargo until 2 pm Eastern Time on the date of publication. For more information, please contact onepress@plos.org.

Kind regards,

Omid Beiki, MD, PhD, MSc

Academic Editor

PLOS One

Additional Editor Comments (optional):

Reviewers' comments:

Reviewer's Responses to Questions

**Comments to the Author**

Reviewer #1: All comments have been addressed

**********

2. Is the manuscript technically sound, and do the data support the conclusions?

Reviewer #1: Yes

**********

3. Has the statistical analysis been performed appropriately and rigorously?

Reviewer #1: Yes

**********

4. Have the authors made all data underlying the findings in their manuscript fully available?

The PLOS Data policy

Reviewer #1: Yes

**********

5. Is the manuscript presented in an intelligible fashion and written in standard English?

Reviewer #1: Yes

**********

Reviewer #1: thank you for addressing all of the comments and making the requested revisions. the manuscript has been improved and i have no further concerns. i recommend acceptance.

**********

what does this mean?). If published, this will include your full peer review and any attached files.

If you choose “no”, your identity will remain anonymous but your review may still be made public.

**Do you want your identity to be public for this peer review?**  For information about this choice, including consent withdrawal, please see our Privacy Policy

Reviewer #1: No

**********

# Editor report: Acceptance letter

**DOI:** 10.1371/journal.pone.0357824.r006

**Authors:** Omid Beiki

PONE-D-26-00877R2

PLOS One

Dear Dr. Agrawal,

I'm pleased to inform you that your manuscript has been deemed suitable for publication in PLOS One. Congratulations! Your manuscript is now being handed over to our production team.

At this stage, our production department will prepare your paper for publication. This includes ensuring the following:

* All references, tables, and figures are properly cited

* All relevant supporting information is included in the manuscript submission,

* There are no issues that prevent the paper from being properly typeset

You will receive further instructions from the production team, including instructions on how to review your proof when it is ready. Please keep in mind that we are working through a large volume of accepted articles, so please give us a few days to review your paper and let you know the next and final steps.

Lastly, if your institution or institutions have a press office, please let them know about your upcoming paper now to help maximize its impact. If they'll be preparing press materials, please inform our press team within the next 48 hours. Your manuscript will remain under strict press embargo until 2 pm Eastern Time on the date of publication. For more information, please contact onepress@plos.org.

You will receive an invoice from PLOS for your publication fee after your manuscript has reached the completed accept phase. If you receive an email requesting payment before acceptance or for any other service, this may be a phishing scheme. Learn how to identify phishing emails and protect your accounts at https://explore.plos.org/phishing.

If we can help with anything else, please email us at customercare@plos.org.

Thank you for submitting your work to PLOS One and supporting open access.

Kind regards,

PLOS One Editorial Office Staff

on behalf of

Dr. Omid Beiki

Academic Editor

PLOS One
